# Bioengineered Silver Nanoparticles: Next-Generation Biogenic Synthesis Strategies for Precision Biomedical Applications

**DOI:** 10.3390/bioengineering13050587

**Published:** 2026-05-20

**Authors:** Mythileeswari Lakshmikanthan, Sakthivel Muthu, Indra Neel Pulidindi

**Affiliations:** 1 Marine Nanobiotechnology Laboratory, Department of Research, Saveetha College of Nursing (SCON), Saveetha Institute of Medical and Technical Sciences (SIMATS), Saveetha University, Thandalam, Chennai 602105, Tamil Nadu, India; 2 Natural Biomedicine Laboratory, Department of Dermatology, Saveetha Medical College and Hospital (SMCH), Saveetha Institute of Medical and Technical Sciences (SIMATS), Saveetha University, Thandalam, Chennai 602105, Tamil Nadu, India; 3 Department of ENT, Saveetha Medical College and Hospital (SMCH), Saveetha Institute of Medical and Technical Sciences (SIMATS), Saveetha University, Thandalam, Chennai 602105, Tamil Nadu, India; indraneelp.smc@saveetha.com

**Keywords:** biogenic AgNPs, green nanotechnology, bioengineered NPs synthesis, precision nanomedicine, biomedical application

## Abstract

Silver nanoparticles (AgNPs) have attracted substantial scientific interest in biomedical research owing to their unique physicochemical characteristics, broad-spectrum antimicrobial activity, plasmonic properties, and therapeutic versatility. Although conventional physicochemical synthesis methods enable controlled NPs fabrication, their dependence on hazardous reagents, elevated energy input, and environmentally detrimental processing conditions has stimulated the development of sustainable biogenic alternatives. Biological synthesis utilizing plants, microorganisms, fungi, algae, and purified biomolecules has emerged as an eco-friendly and bio-compatible strategy for AgNP fabrication, enabling simultaneous reduction, stabilization, and intrinsic biofunctionalization of NPs. However, traditional biogenic synthesis remains constrained by limited mechanistic understanding, poor batch reproducibility, inadequate control over physicochemical properties, and challenges in large-scale manufacturing. Recent advances in bioengineering have transformed this field through the integration of metabolic engineering, synthetic biology, microfluidic-assisted synthesis, artificial intelligence-guided process optimization, and continuous-flow biomanufacturing, collectively enabling precision fabrication of biogenic AgNPs with enhanced uniformity, scalability, and functional tunability. Furthermore, strategic surface engineering and functionalization have expanded the applicability of biogenic AgNPs across targeted anticancer therapy, antimicrobial intervention, wound healing, regenerative medicine, drug delivery, and theranostic imaging. Despite these advancements, critical challenges remain regarding nano–bio interactions, toxicological safety, regulatory compliance, and translational scalability. Unlike conventional reviews focused primarily on green synthesis approaches, this review critically highlights emerging bioengineering paradigms that enable programmable, scalable, and precision-controlled biogenic AgNP fabrication. This review comprehensively examines next-generation paradigms and strategies for AgNPs biosynthesis, elucidates the molecular mechanisms governing their formation, highlights emerging functionalization and biomedical application paradigms, and discusses current translational barriers. Forming biogenic composites of AgNPs and heteroatom doped carbon nanodots needs intense research in near future.

## 1. Introduction

Integrated areas of nanotechnology and biomedicine emerge as significant, since silver nanoparticles (AgNPs) are demonstrated in visible light with unique physiochemical properties, tunable localized surface plasmon resonance (LSPR), better surface-area- to-volume ratio, and high junction-conducting capacity with broad-spectrum biological activities, making it amongst the best viable noble-metal nanomaterials in modern nanobiotechnology [1]. Such inherent properties have allowed their use in many different biomedical applications such as antimicrobial therapeutics, biosensing, wound repair and management systems, targeted drug delivery, multi-modal molecular imaging modalities, and cancer nanotheranostics [2,3]. In addition, growing attention to AgNPs arises from their extensive biological activity against multidrug-resistant pathogens by causing oxidative stress-mediated apoptosis in tumor cells [4]. The traditional conventional physicochemical approaches for the synthesis of AgNPs, including chemical reduction, electrochemical deposition, thermal decomposition, laser ablation, and microwave-assisted fabrication, subsume a core of fabrication sequences that have allowed complex control over NPs morphology and colloidal stabilization over recent decades [5]. By contrast, such protocols usually rely on toxic reducing agents, organic solvents, and high-energy input or reaction conditions with dire environmental, toxicological, and economic merits. Moreover, trace chemical impurities from such synthetic approaches can greatly impact NPs’ biocompatibility and further limit their readiness to advance into clinical/biomedical applications [6,7].

These reasons have made biogenic or “green” synthesis pathways a key focus as sustainable methods for AgNP production [8]. Biogenic NPs can be synthesized utilizing the reductive as well as stabilizing abilities of naturally occurring biomolecules derived from plants, microorganisms (bacteria), fungi, algae, enzymes, or other biological systems to reduce silver ions (Ag^+^) in solution into elemental silver nanostructures (Ag^0^) [9,10]. Use of biological matrices not only eliminates the need for toxic reagents but also guarantees that, owing to surface-associated phytochemicals, proteins, polysaccharides, and secondary metabolites, intrinsic biofunctionalization will result, which enhances the stability and therapeutic performance of NPs [11]. Nevertheless, although the literature base on green synthesis of AgNPs is large and growing rapidly, most reports remain broadly descriptive and exploit relatively simple biosynthesis pathways or applications, while a much smaller number of resources have gone into elucidating mechanisms, high-throughput syntheses, or scaled translation [12,13]. Compounding this limitation, biogenic AgNPs in their totality suffer from a lack of control over some key parameters (i.e., particle size distribution, morphology, surface chemistry, batch reproducibility, and physicochemical uniformity across biosynthetic platforms). This restriction underlines the necessity of a more complex alternative for conventional green synthesis paradigms, i.e., a bioengineered NPs-building technique [14,15,16].

Emerging breakthroughs in synthetic biology, metabolic engineering, microfluidics, machine-learning guided optimization, and continuous-flow biomanufacturing catalyzed the transition of bioengineered AgNP synthesis frameworks to a next-generation paradigm [17]. These unique methodologies also permit appropriate fine-tuning of biosynthetic pathways, improved reaction kinetics, better monodispersity, and industrial production in homogeneous conditions [18]. In addition, this integration of rational surface engineering with targeted functionalization has opened the unique possibility of employing these biogenic AgNPs within precision medicine, whereby engineered nanostructures may be designed for site-specific delivery, stimuli-responsive release, and multimodal therapeutic intervention [19].

Unlike previous reviews that predominantly summarize conventional green synthesis methodologies and general biomedical applications of AgNPs, the present review specifically focuses on the emergence of next-generation bioengineered biosynthesis platforms that integrate metabolic engineering, synthetic biology, microfluidic-assisted fabrication, artificial intelligence/machine learning (AI/ML)-guided optimization, and continuous-flow biomanufacturing strategies. Furthermore, this review critically evaluates the mechanistic limitations of traditional biogenic synthesis systems, including poor reproducibility, physicochemical heterogeneity, limited scalability, and translational bottlenecks. Particular emphasis is placed on precision surface engineering, programmable nanofabrication, and future translational pathways toward clinically relevant and industrially scalable AgNP production systems. Therefore, this review provides a forward-looking perspective that extends beyond descriptive green synthesis and highlights the transition toward precision biofabrication and intelligent nanomanufacturing paradigms.

## 2. Advancements in the Biogenic Synthesis of AgNPs

### 2.1. Traditional Chemical and Physical Methods

The first methods used for the synthesis of AgNPs were mainly based on physicochemical concepts and employed reductive chemistry, as well as energy-consuming physical approaches to produce nanoscale silver structures with specified morphology [20]. Chemical reduction, still one of the most utilized traditional methods, is a procedure where silver salts, for example, silver nitrate, are reduced in the presence of strong reducing agents such as sodium borohydride, hydrazine hydrate, among others like trisodium citrate or ethylene glycol [21]. The rigor with which these protocols allow for control over NPs nucleation and growth kinetics results from careful tuning of reagent concentration, pH, temperature, and the type of stabilizing surfactants used. Many physical methods (laser ablation, thermal evaporation condensation, microwave irradiation, arc discharge, and ultrasonic spray pyrolysis) also allow production of AgNPs with solid size-distribution control and high purity levels, but require special processing environments [21,22].

While reproducible and precise, the methods come at a high cost. However, the use of harmful reducing agents and organic solvents creates toxicological liabilities that jeopardize downstream biomedical compatibility [23]. Secondly, the necessity of receiving high thermal or radiative energy input increases both production expenses and reduces the environmental sustainability [24,25]. The physicochemical residues that remain adsorbed on NPs surfaces can modulate cellular responses, drive immunogenicity, and hamper regulatory approval as therapeutics. Therefore, as traditional synthesis platforms have made important strides in nanomaterial science, it has been the translational limitations of these platforms into advanced applications that motivated the search for more sustainable fabrication routes [26].

### 2.2. Transition to Green/Biogenic Synthesis

This transition from conventional synthesis methods to more environmentally friendly green or biogenic pathways stems from the increasing need for environmentally innocent and biologically compatible nanomanufacturing processes [26,27]. The principle behind green synthesis is the use of biological systems or biomolecules derived from nature to be employed as reducing, capping, and stabilizing agents during NPs fabrication. This paradigm shifts into alignment with the green chemistry tenet of minimizing hazardous waste, limiting energy consumption, and substituting drug-classifying toxic reagents for naturally occurring, renewable biological substrates [28,29,30].

**Figure 1:**
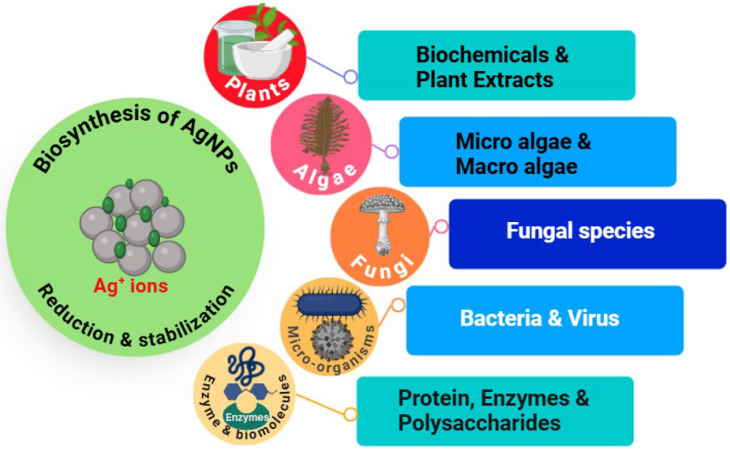
Biological sources, including plants, algae, fungi, microorganisms, and biomolecules, mediate the reduction of Ag^+^ ions to AgNPs through simultaneous reduction and stabilization processes.

Early demonstrations of AgNP biosynthesis relied on crude plant extracts with high phytoconstituent content, including polyphenols, flavonoids, terpenoids, alkaloids, and sugars that can reduce silver ions [31,32,33]. Later research broadened this biosynthetic group to include bacteria, fungi, yeast, microalgae, and cyanobacteria using an intracellular or extracellular enzymatic machinery for metal bioreduction. These biological factors have the innate property of binding to metal ions, and such spontaneous affinity has enabled the use of these biological materials in one-pot synthesis systems where reduction occurs, including stabilization, commonly giving particles that are colloidally stable or offer increased bioactivity [34,35]. In this context, biogenic synthesis brings with it the concept of biofunctionalization of NPs intrinsic to their biosynthesis, wherein residual biomolecular residues physically adsorbed onto the surface of biogenic NPs impart additional pharmacological, colloidal, and targeting properties. Surface coronas of this kind have been demonstrated to influence cellular uptake, reduce aggregation, and improve therapeutic interactions, thereby endowing biogenic AgNPs with features not seen amongst their chemical counterparts [15,36] (Figure 1).

### 2.3. Traditional Biogenic AgNPs Production: Shortcomings

Despite the environmental and biomedical benefits of biogenic aspects, traditional biogenic synthesis systems remain limited in several fundamental aspects, preventing reproducibility, mechanistic control, and scaling for industrial applications [37]. One major difficulty is the compositional diversity of biological extracts, which consist of complex and largely uncharacterized mixtures of reducing and stabilizing biomolecules. NPs nucleation kinetics and final physicochemical properties may differ greatly because of variability in plant metabolite content, microbial growth conditions, extraction procedures, seasonal fluctuations, and geographic origin [38].

Consequently, biosynthesized AgNPs generally possess a wide range of sizes, variable shapes, and inter-batch variability regarding colloidal stability and biological activity. In addition, mechanistic pathways controlling bioreduction, nucleation, growth, and stabilization remain poorly understood for many biological systems, limiting rational optimization of synthesis conditions. Tunable control over the degree of particle anisotropy, crystallinity, or surface chemistry remains a major hurdle for applications in high-precision NPs standardization, especially in clinical nanomedicine [39]. Further restrictions include poor synthetic yields, reaction times that are not fit for a production environment, and downstream purification challenges, as well as complications involved with the translation of lab-scale biosynthetic protocols to industrial-scale quantities. The combined limitations all highlight the importance of developing advanced biosynthetic systems that offer greater precision and controllability [40,41].

### 2.4. The Need for Next-Generation Bioengineered Approaches

To overcome the constraints associated with traditional biogenic synthesis, the field has been gradually shifting towards bioengineered next-generation strategies that merge fundamentals of systems biology, metabolic engineering, synthetic biology output amplification, process intensification, and computational optimization [42]. These sophisticated frameworks are designed to convert natural biological synthesis from passive methods into programmed nanomanufacturing systems for AgNPs with desired physicochemical characteristics and enhanced reproducibility. By recruiting pathways to quantify the P450 family of enzymes, we can better engineer time for metabolic hosts to respond to any intracellular properties such as reductase expression, cofactor availability, and metal ion transport pathways by enhancing each step (e.g., gene clustering), increasing efficiency and NPs yield in a targeted manner, and metabolic engineering [43]. Synthetic biology strategies also enable rational design of engineered cellular chassis capable of programmed stimuli-dependent NPs biosynthesis using gene circuits. Microfluidic-assisted synthesis platforms simultaneously provide a manner for one-pot control over reaction kinetics, reagent mixing, and nucleation dynamics in continuous-flow mode, thereby enabling uniform NPs production at scale output [44,45].

Introduction of artificial intelligence and machine-learning algorithms into biosynthetic optimization has also become a ground-breaking approach to predict best synthesis conditions from multi-dimensional experimental data sets [46,47]. Computational methods can help speed up parameter screening, reduce empirical trial-and-error experiments, and provide predictive control over the morphology and functionality of NPs [48]. Together, these innovations mark a transition from trial-and-error green synthesis towards directed biofabrication of silver nanostructures. The bioengineered AgNP synthesis thus has great potential to associate state-of-the-art laboratory-scale biosynthesis with leading-edge nanomedicine manufacturing translation [49].

## 3. Nanobiological Factories for Sophisticated AgNPs Biogenic Synthesis

### 3.1. Plant-Derived Phytochemical-Mediated Synthesis

Plant-mediated biosynthesis is one of the most investigated points for the green synthesis of AgNPs due to its simplicity, reaction kinetics, and a wide range of phytochemicals [50]. In this manner, aqueous or solvent-based plant extracts function as both reducing and stabilising matrices, activating a diverse arsenal of bioactive metabolites (polyphenols, flavonoids, terpenoids, alkaloids, tannins, proteins such as enzymes, cofactor-like molecules, reducing sugars, and organic acids) that mediate the reduction of Ag^+^ ions to Ag^0^ nanostructures [51]. Hydroxyl, carbonyl, amine, and carboxyl functional groups of these biomolecules actively engaged in the formation of covalent bonds by either donating electrons or initiating nucleation, as well as those involved in affecting surface passivation processes, were suggested to influence NPs’ morphology and colloidal stability [52,53].

Particularly, the composition of plant extracts largely determines the physicochemical characteristics (hydrodynamic size and diameter, anisotropy, zeta potential, and crystallinity) of synthesized NPs. Changes in species selection, plant organ used, developmental stage, geographical origin, and separation method can have a significant effect on metabolite compositions and therefore modify NP formation kinetics [54]. While plant-mediated synthesis is appealing because of its availability and the cost factor, one critical barrier to reproducibility and mechanistic standardization remains the enigma of inbuilt compositional heterogeneity of botanical extracts. However, plant-based AgNPs usually have additional advantages for biomedical applications as bioactive phytoconstituents can get anchored on their surface and may exert antioxidant, anti-inflammatory, antimicrobial, and anticancer activities. Thus, phytochemical-mediated synthesis remains a useful and generalisable biosynthetic platform with potential for exploratory biomedical uses, as well as the construction of new classes of multifunctional nanotherapeutics [55].

### 3.2. Bacterial Nanofactories

Bacterial systems have been proven to be promising microbial nanofactories for AgNP biosynthesis due to their rapid growth, genetic modification flexibility, metabolic versatility, and ability to produce both intracellular and extracellular NPs [56]. Over time, many Gram-positive and Gram-negative bacterial groups developed inherent resistance to metal ion toxicity by employing native enzymatic systems capable of biotransforming silver ions into elemental silver nanostructures. Key enzymes, including nitrate reductase, NADH-dependent reductases, hydrogenases, and electron shuttle proteins, are assumed to be involved in bacterial reduction of Ag^+^ ions [57].

Intracellular biosynthesis cells take up silver ions through membrane-associated transport systems and reduce them enzymatically in the cytoplasm or periplasmic space, frequently producing relatively monodispersed NPs. Fortunately, biosynthesis can be based on two different mechanisms: in contrast to intracellular synthesis, where silver ions are reduced by secreted enzymes, peptides, and exopolysaccharides initiated from the medium (extracellular), allowing a straightforward recovery and purification of NPs [58]. Reduced downstream processing requirements make the extracellular route especially favorable for scalable manufacturing. Bacterial systems are amenable to metabolic engineering and synthetic biology, making them practical candidates for programmable NPs biosynthesis. Through genetic modification approaches, it may be possible to increase reductase expression or improve silver ion transport and control extracellular polymer release to provide improved tunability over NPs size, yield, and surface properties. Still, the threat of endotoxin contamination, biosafety implications, and strain-dependent variability are considerable obstacles for implementation [59].

### 3.3. Fungal-Mediated AgNP Biosynthesis

Silvery NPs biosynthesis was realized in the fungal nanofactories, which gathered considerable interest as they are known for extraordinary secretory capability, remarkable production of extracellular enzymes, and the ability to withstand higher concentrations of metal ions. Filamentous fungi and yeasts produce large amounts of mycelial biomass and, with the aid of their extracellular protein secretion systems, can rapidly reduce and immobilize silver ions to provide monodispersed NPs in solutions, often with better qualities than those produced by bacterial systems [60]. Nitrate reductase, laccase, quinone reductase, and other oxidoreductases associated with the processes of electron transfer and silver ion reduction are the key fungal enzymes for AgNP biosynthesis. Furthermore, because fungal secretomes are rich in natural capping agents (the family of polysaccharides, proteins, amino acids, and secondary metabolites), they can seal NPs to improve their stability and biocompatibility. Extracellular fungal biosynthesis is of particular benefit since it allows convenient harvesting of NPs from the culture supernatants, circumventing a cellular disruption step [61,62].

This advantage could also mean a dramatic improvement in terms of scalability compared with more commonly used regional plant-based systems through fermentation-based cultivation of fungi (this is particularly true when considering the relative ease and feasibility with which fungal growth can be controlled). On the other hand, long cultivation times, vulnerability to contamination, and fluctuation in metabolite secretion patterns may be obstacles for process optimization. Despite these challenges, fungi still stand out as one of the most lucrative biological platforms for industrial-scale AgNP biomanufacturing [63,64].

### 3.4. Algal and Cyanobacterial Platforms

Photosynthetic microorganisms such as microalgae, macroalgae, and cyanobacteria have recently been proven to be promising and highly efficient platforms for AgNP biosynthesis owing to their rich repertoire of reductive pigments, sulfated polysaccharides, proteins, phenolic compounds, and antioxidant metabolites, which lead to high potential reduction [65]. Algal biomass and extracts contain inherently strong metal-chelating and reducing abilities, which expedite the reduction of silver ions under ambient conditions. Among those marine derivatives, marine macroalgae have been attracting more attention and are reported to contain several sulfated polysaccharides, including fucoidan, carrageenan, and ulvan, which not only induce the NPs synthesis but also increase their colloidal stability and biological activities. Similarly, cyanobacteria release extracellular polymeric substances and redox-active pigments, including phycobiliproteins and derivatives of chlorophyll, leading to NPs formation [66,67,68].

Algal and cyanobacterial synthesis platforms have several unique benefits, such as renewable biomass production, low nutrient demand, and compatibility with carbon-neutral cultivation systems. In addition, the algal metabolic-derived biomolecular coating of biosynthesized AgNPs may also lend certain immunomodulatory and anticancer properties. But aside from this, scalability would still rely on biomass cultivation, harvesting, and extract standardization [69].

### 3.5. Enzyme-Directed and Cell-Free Biosynthesis

Enzyme-directed and cell-free biosynthesis is a more sophisticated form of biological NPs production, using isolated enzymes or purified biomolecular fractions, for example, instead of a whole-cell or crude extract system [70]. Compared to the classical biosynthetic approach, this strategy provides improved mechanistic control, reduced biological variation, and better standardization. Most purified reductive enzymes, including nitrate reductase, glucose oxidase, laccase, and peroxidases, can catalyze Ag^+^ reduction under well-controlled reaction conditions, which affords more predictable tumor nucleation and growth kinetics. Similarly, they can act as a reducing and templating agent for edge-shape-controlled nanostructures with surface chemistry-specific features created by isolated peptides, proteins, and polysaccharides [71].

**Figure 2:**
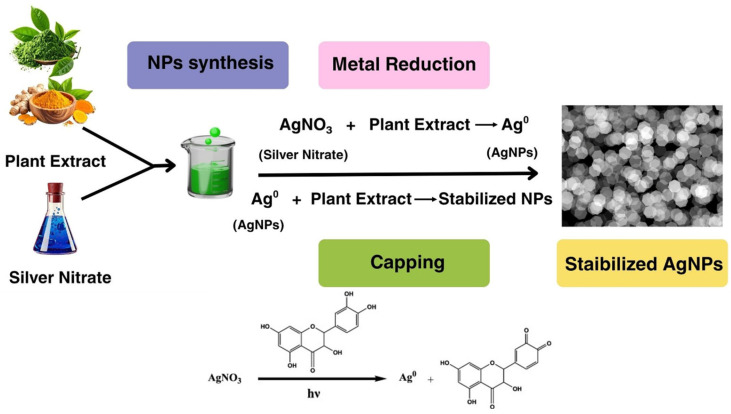
Green synthesis of AgNPs using plant extracts, where phytochemicals reduce AgNO_3_ to Ag^0^ (metal reduction) and simultaneously act as capping agents to stabilize the formed nanoparticles.

Cell-free biosynthesis overcomes several challenges inherent in whole-cell systems, such as risk for contamination, difficulties recovering synthesized products from within cells, and cellular heterogeneity in metabolic behavior [71]. Additionally, purified biomolecules can be used to mechanistically dissect pathways of NPs formation and enable rational process engineering. Yet, the expensive enzyme purification and inherent limited catalytic stability still limit its large-scale application. Notwithstanding these hurdles, enzymatic-based strategies represent a highly promising approach toward precision biofabrication of AgNPs and act as an essential link between empirical green synthesis and entirely engineered NPs manufacturing systems [72,73,74] (Figure 2 and Table 1).

## 4. The Role of Molecular Mechanisms in Biogenic AgNP Formation

### 4.1. Pathways of Reduction of Ag^+^ to Ag^0^

The formation of AgNPs through biogenic synthesis starts with the reduction of ionic silver (Ag^+^) to elemental metallic silver (Ag^0^) and is underpinned by intricate redox interactions between Ag ions and electron donor molecules. While reduction in biological systems can occur via enzymatic catalysis, through direct electron transfer from redox-active metabolites or the synergistic behavior of multiple biomolecular constituents, this is not the answer. The ability of many naturally occurring biomolecules bearing hydroxyl, carbonyl, amine, sulfhydryl, and carboxyl functional groups to exercise significant redox potential with the highly redox-active Ag^+^ ion makes its reduction a thermodynamically favorable reaction [75].

In plant-mediated systems, polyphenolic compounds and flavonoids often serve as major electron donors through hydroxyl moiety oxidation to quinone intermediates while reducing Ag^+^ ions to a stable form of metallic silver (Ag^0^). In microbial systems, electron transfer from cofactors (e.g., NADH/NADPH) to silver ions is catalyzed by intracellular or extracellular oxidoreductases either directly or via intermediate electron transport mediators. These compact zero-valent silver atoms then cluster together to nanoscale nuclei and trigger the formation of NPs. pH, temperature, precursor concentration, redox potential of the biological matrix, and a variety of catalytic cofactors strongly influence the rate and efficiency of this reduction [76,77].

### 4.2. Contributions of Secondary Metabolites and Redox Enzymes

Biogenesis of AgNPs is primarily mediated by secondary metabolites and redox-active enzymes. Phytochemical-mediated synthesis is broadly divided into six major components: flavonoids, phenolic acids, tannins, terpenoids, alkaloids, and reducing sugars, which are involved in the chelation of metal ions and transferring electrons. The redox-activated functional groups and conjugated aromatic systems of these metabolites can stabilize transient silver intermediates on common reduction pathways [78]. In microbial and fungal biosynthesis, enzymatic reduction makes a more significant contribution. This is mostly due to the implication of nitrate reductase, which has been proposed as a key catalyst in AgNP biosynthesis through electron transfer from NADH to silver ions. Several other enzymes, such as laccases, quinone reductases, hydrogenases, and dehydrogenases, have also shown a correlation with silver bioreduction in multiple microbial phyla [79]. Such enzymatic pathways facilitate the reduction of kinetics but also may play a role in influencing NPs’ shape by promoting fluctuations in the supersaturation of silver atoms locally. Significantly, the combination of enzymatic and non-enzymatic reducing elements frequently develops cooperative biosynthetic microenvironments for NPs where various compounds simultaneously participate in nucleation, growth, and stabilization. This biochemical complexity helps clarify the improved functionalities and bioactivities we see in biogenic AgNPs plot [70,72].

### 4.3. Nucleation and Growth Kinetics

Following the reduction of Ag^+^ to Ag^0^, NPs formation proceeds through a nucleation-growth mechanism analogous to classical colloidal crystallization processes. Initial supersaturation of reduced silver atoms leads to spontaneous nucleation, during which small clusters of Ag^0^ atoms coalesce to form thermodynamically stable nuclei [80]. Once the critical nucleus size is attained, subsequent silver atom deposition drives anisotropic or isotropic NPs growth depending on the local biochemical environment. The kinetics of nucleation and growth critically determine the final NPs’ size distribution, morphology, crystallographic structure, and colloidal monodispersity [81]. Rapid reduction rates generally favor burst nucleation and formation of smaller, more uniform NPs due to simultaneous generation of numerous nuclei. Conversely, slower reduction kinetics promote prolonged growth phases, often yielding larger and more polydisperse nanostructures [82].

Biological macromolecules present within the reaction milieu may selectively adsorb onto crystallographic facets of nascent silver nuclei, thereby modulating directional growth and promoting the formation of anisotropic morphologies such as rods, triangles, cubes, or prisms. Parameters, including pH, ionic strength, temperature, precursor concentration, and biomolecule-to-metal ratio, exert a substantial influence over these nucleation-growth dynamics and thus represent critical determinants of NPs architecture [78,83].

### 4.4. Natural Capping and Stabilization Processes

One of the key characteristics of biogenic AgNP synthesis is self-capping by a biomolecule surrounding nanomaterial surfaces. This spontaneously occurring capping layer consists of adsorbed proteins, polysaccharides, polyphenols, lipids, peptides, and other biomolecules from the biological synthesis matrix [84]. The capping layer functions to stabilize NPs by steric hindrance, electrostatic repulsion, or hydration shell formation, avoiding agglomeration while providing stability and extended colloidal longevity [85]. The strong anchoring of the capping biomolecules to AgNPs comes from coordination bonding, electrostatic attraction, or van der Waals interactions between functional groups such as hydroxyl, amine, carboxyl, phosphate, and sulfhydryl moieties. More than just an intended stabilizing feature, they play a large role in determining the surface charge, hydrodynamic size, protein adsorption behavior, and cellular interactions of their encapsulated NPs cargo [86]. Finally, it is an important advantage of the in-situ bio-capping of AgNPs that, compared to chemically stabilized NPs, they can obtain a higher biological compatibility. The surface-bound phytochemicals, or even the microbial biomolecules (including proteins, polysaccharides), provide intrinsic antioxidant, anti-inflammatory, targeting, or therapeutic properties, which increase biomedical efficacy. Nevertheless, inappropriate and/or uncontrolled capsaicin capping may further limit reproducibility and challenge the mechanistic interpretation of biological responses [82,87].

### 4.5. Bio-Corona Assembly and Surface Functional Identity

Biosynthesized AgNPs dynamically adsorb a spectrum of biomolecules from the surrounding milieu upon exposure to biological fluids or physiological environments, forming biologically laden surfaces with a secondary biomolecular corona known as the bio-corona. This corona is acquired and coats the pre-existing biosynthetic capping layer, providing a novel delineation of NPs biological identity during cellular and systemic interaction. The physical and chemical characteristics (size, charge, hydrophobicity, surface roughness, pre-existing biomolecular coating) of the NPs shape their bio-corona composition and architecture. The totality of adsorbed proteins, lipids, metabolites, and nucleic acids collectively dictates NPs recognition by immune cells, the receptor-mediated uptake pathways, biodistribution profiles, and intracellular trafficking mechanisms [88,89].

The pre-existing natural capping layer surrounding biogenic AgNPs may significantly modify the key attributes of bio-corona formation compared to chemically synthesized NPs, potentially improving stealth behavior, lowering non-specific protein adsorption, and promoting selective cell interaction. This makes it critical to understand the relationship between the biosynthetic interplay of capping and acquired bio-corona in predicting NPs pharmacokinetics, therapeutic efficacy, and toxicology. Mechanistically acting pathways controlling Ag^+^ reduction, nucleation, growth, stabilization, and interaction with the bio-corona define together the molecular basis of the biogenic synthesis of AgNPs. A thorough explanation of these pathways is inevitable to progress from biosynthesis-based empirical approaches to rationally designed and clinically applicable NPs preparation [90] (Table 2).

## 5. Advanced Bioengineering Strategies for Controlled AgNPs Fabrication

### 5.1. Metabolic Engineering of Microbial Nanofactories

Metabolic engineering has paved a novel smart strategy for improving the biosynthetic efficiency and precision of microbial nanofactories used in AgNPs production. Using rational rewiring of intracellular metabolic pathways, we increase reductive flux, optimize cofactor regeneration, and improve silver ion detoxification systems to boost NPs yield and uniformity [93]. Moreover, genetic overexpression of a few oxidoreductases like nitrate reductase, quinone reductase, and NADH-dependent dehydrogenases has been reported to increase Ag^+^ reduction kinetics and enable homogenous nucleation of NPs. At the same time, regulation of intracellular glutathione metabolism and metal transport proteins might not only boost cellular silver stress tolerance but also allow for higher precursor loading and increased biosynthetic productivity [92,94]. Several microbial systems have shown enhanced AgNP biosynthesis through modulation of reductive and metal-resistance pathways. Engineered strains of *Escherichia coli* and *Pseudomonas aeruginosa* have been explored for increased expression of nitrate reductase, NADH-dependent reductases, and glutathione-mediated detoxification systems to improve Ag^+^ reduction and silver tolerance [95,96]. Similarly, *Bacillus subtilis* has been investigated for enhanced extracellular polymeric substance secretion to improve nanoparticle stabilization [97]. Metal transport systems such as ABC transporters and SilE/SilP-associated silver efflux pumps may further regulate intracellular silver homeostasis and biosynthetic efficiency, enabling improved control over nanoparticle yield and physicochemical uniformity [98].

Apart from reductive modification via the means of metabolic engineering, it empowers regulation of secretion systems responsible for extracellular polymeric substance (EPS) deposition, as well as controlling peptide biosynthesis or exopolysaccharide composition, which effectively up- and down-regulate any desired property influencing NPs stabilization and surface chemistry [99]. These programmable, naturally occurring microbial systems, conventional optogenetic approaches inspired by animal and plant circadian rhythms, transform scientific biosynthetic chassis into directed biosynthetic chassis with enhanced reproducibility and customizable properties (e.g., optimized physicochemical attributes of AgNPs) [100]. Despite promising advances, metabolic engineering approaches remain constrained by genetic instability, intracellular metal toxicity, metabolic burden, and limited availability of standardized engineered strains for industrial nanomanufacturing.

### 5.2. Synthetic Biology Approaches

Synthetic biology takes traditional metabolic engineering a step further, allowing the construction of programmable and modular genetic circuits for significant control over biosynthesis of NPs [101]. Engineered biosynthetic modules, synthetic promoters, inducible gene switches, and regulatory feedback loops can be incorporated into a microbial or cellular platform to initiate NPs synthesis, given defined environmental or molecular stimuli [102]. Programmable gene circuits might be exploited to temporally constrain reductase expression, crosstalk silver ion uptake with intracellular detoxification pathways, and couple nucleation of the NPs with release of stabilizing biomolecules. These control systems enable dynamic tuning of NPs biosynthesis to minimize heterogeneity and improve process standardization. Moreover, with the capacity to integrate non-native biosynthetic pathways and heterologous gene expression systems into the same host, synthetic biology also provides opportunities for customized hosts capable of more efficient metal reduction or specific capping activity. While synthetic biology is at an early stage for the fabrication of NPs, it has great potential to enable genetically encoded programmability and deployment of so-called “living nanofactories” that can autonomously generate AgNPs [103]. Emerging synthetic biology approaches use inducible promoters, quorum-sensing circuits, and synthetic regulatory modules to control reductase expression and AgNP biosynthesis. Heterologous expression of nitrate reductase and quinone reductase systems has been explored to improve biosynthetic precision and nanoparticle formation. However, challenges related to genetic stability, biosafety, and industrial scalability still limit practical applications. Current synthetic biology strategies for AgNP biosynthesis remain largely proof-of-concept, and substantial challenges persist regarding biosafety, regulatory approval, genetic containment, and large-scale deployment of engineered living nanofactories [104,105].

### 5.3. Microfluidic-Assisted Biogenic Synthesis

Microfluidic-based biosynthesis is a big leap in controlled biogenic AgNPs synthesis, through substantially specific modulation over the microenvironments of reaction stages within miniaturized continuous-flow devices [106]. Microfluidic platforms allow for laminar flow-based mixing, precise reagent dosing, rapid thermal equilibration, and spatially resolved control of nucleation and growth kinetics in a different way compared to bulk batch synthesis [107]. The microscale confinement improves mass transfer and keeps the concentration gradient over a small distance, while it also favors homogeneous supersaturation of the solution to nucleate NPs. The use of microfluidic completeness in biosynthesis, therefore, provides a convenient approach to produce AgNPs with narrower size distributions, superior monodispersity, and better reproducibility compared to conventional flask-based methods [108]. Moreover, parameters of reaction, i.e., residence time, flow rate, precursor concentration, and temperature in the reactor, are dynamically controllable (in real time) to yield the desired characteristics. Native biological extracts, isolated enzymes, or engineered microbial secretomes each promise a scalable and standardizable path forward for continuous biogenic AgNP production on industrially relevant time scales [109]. Microfluidic-assisted synthesis improves reaction reproducibility through precise control of residence time, reagent mixing, and thermal gradients. Integration of inline UV–Vis spectroscopy and DLS monitoring enables real-time process control during nanoparticle fabrication. However, device fouling, limited throughput, purification complexity, and scale-up challenges remain major limitations. Although microfluidic systems provide exceptional control over nucleation kinetics and nanoparticle uniformity, practical limitations, including device fouling, fabrication complexity, low throughput, and integration with large-scale manufacturing, remain major translational obstacles [106].

### 5.4. AI/ML-Guided Process Optimization

Recently, AI and ML have been applied to facilitate the optimization of complex biosynthesis processes starting from NPs [110,111]. Many of the RHS, which include several interdependent parameters such as precursor concentration, biological extract composition, pH, temperature, reaction time/residence time/mass density, and mixing conditions, influence AgNP fabrication through nonlinear interconnection and are poorly predictive [112]. Multidimensional experimental datasets can thus be used to train machine learning algorithms, which uncover hidden relationships between the experimental parameters, predict the results of synthesis trials, and propose optimal reaction conditions for target NPs characteristics. The application of methods such as artificial neural networks, support vector machines, random forest regression, and Bayesian optimization has proven useful for the prediction of NPs size, morphology, zeta potential, and yield based on biosynthetic input variables [113]. AI-driven optimization abolishes a significant part of empirical trial-and-error experimentation, thus speeding up process development and improving reproducibility [114]. Then, one could envision automated microfluidic or bioreactor platforms to be integrated with ML algorithms for autonomous closed-loop systems for NP synthesis in the next generation of manufacturing paradigms [115]. Despite growing interest in AI/ML-guided optimization, current predictive models are limited by small and non-standardized biosynthetic datasets. Variability in biological extracts and synthesis conditions increases the risk of overfitting and poor reproducibility. Furthermore, external validation across independent biosynthetic systems remains limited, restricting large-scale translational reliability. AI/ML-guided optimization is further limited by insufficient availability of large standardized biosynthetic datasets, variability among biological systems, and challenges associated with model interpretability and experimental reproducibility [116].

### 5.5. Continuous Flow and Scale-Up Biomanufacturing

**Figure 3:**
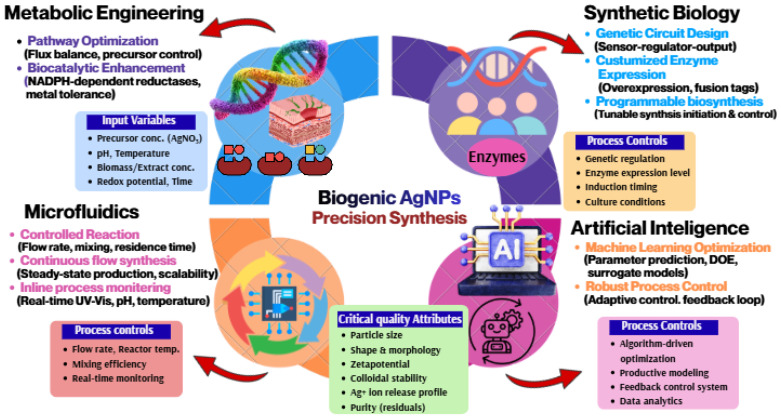
Precision synthesis of biogenic AgNPs using metabolic engineering, synthetic biology, microfluidics, and AI for optimized and controlled nanoparticle production.

Transferring the short-lived laboratory-scale experiment to industrial production of biogenic AgNPs is highly dependent on improved process strategies capable of continuous-flow or scalable biomanufacturing [117,118,119]. Batch-based biosynthesis is often hampered by low reproducibility, mixing efficiency, and heat transfer, with limited assurance of uniform reaction conditions for larger volumes. These limitations can be overcome using continuous-flow bioprocessing, which allows for the uninterrupted introduction of reagents, in-line monitoring of processes, and harvesting of NPs under steady-state conditions [120]. In addition, the precise control over residence time, mixing dynamics, and reaction kinetics that such systems provide results in better product quality from batch to batch, as well as ease of scaling up manufacturing. The online coupling of continuous-flow reactors with inline spectroscopic or particle-sizing analytic devices simplifies the implementation of monitoring and quality assurance based on process analytical technology (PAT). Other upstream approaches may include biosynthesis in bioreactor-integrated systems using engineered microbial or enzymatic platforms, enabling AgNP production at the industrial scale and scalable under Good Manufacturing Practice (GMP)-compatible conditions. However, there are still major hurdles with downstream purification, sterility assurance, long-term reactor stability, and common regulatory standards (Figure 3 and Table 3) [121,122]. Continuous-flow platforms provide improved batch reproducibility, enhanced mass transfer, and consistent nanoparticle quality compared with conventional batch synthesis. Integration with process analytical technology (PAT) enables real-time monitoring of particle size and colloidal stability. However, downstream purification, sterility assurance, and GMP-compliant large-scale manufacturing remain significant challenges [119].

## 6. Biogenic AgNPs Functionalization and Surface Engineering

### 6.1. Ligand Functionalization

Ligand functionalization is a particularly important approach to tailoring the physicochemical and biological properties of biogenic AgNPs by conserving specific molecular moieties on their surface [14]. As the native biomolecular corona of biosynthesized AgNPs contains a multitude of diverse reactive groups, such as hydroxyl, amine, carboxyl, and sulfhydryl functionalities, it is possible to use these on AgNP surfaces to promote increased chemical modifications [90]. Colloidal stabilization of NPs, prolonged circulation in blood, and enhanced targeted receptor-mediated cellular uptake can be achieved by covalently/non-covalently attaching surface ligands such as thiolated polyethylene glycol (PEG), folic acid, peptides, antibodies, aptamers, and small-molecule targeting agents. Ligand engineering also enables tissue- or disease-selective targeting, which enhances therapeutic specificity and reduces off-target cytotoxicity [123,124].

### 6.2. Polymer and Hydrogel Coatings

To enhance the physicochemical stability, controlled release profile, and biocompatibility of biogenic AgNPs, polymeric and hydrogel-based surface coatings are frequently used combined. Overcoating with biocompatible polymers, among others, polyethylene glycol, polyvinyl alcohol, chitosan, alginate, gelatin, and dextran, is used for this purpose, leading to the formation of sterically stabilized protective shells that greatly reduce aggregation and nonspecific adsorption of proteins during their transport in vivo [125,126]. The hydrogel encapsulation also advances the inclusion of AgNPs into 3D biomaterial matrices for long-term local release applications, specifically regarding wound healing, tissue regeneration, and implantable biomedical devices. Such coatings also facilitate the co-delivery of other therapeutic agents, paving the way for multifunctional nanoplatform design [125,127].

### 6.3. Antibody/Aptamer Conjugation

Combining antibodies and aptamers with other biogenic AgNPs allowed the construction of targeted nanoplatforms for specific diagnostic issues or precision therapeutics. Antibody-coated AgNPs harness the recognition of antigens by antibodies to bind specifically to disease-relevant biomarkers, overexpressed receptors, or pathogenic antigens [128]. Likewise, aptamers (short single-stranded oligonucleotides with high-affinity binding capabilities to bind a molecular target) offer an attractive and reversible alternative to antibodies as a possible probe for specific targeting of molecules. These bio-recognition moieties can promote the accumulation of NPs at pathological sites, improve biosensing specificity, and facilitate intracellular delivery of therapeutic payloads [129].

### 6.4. Stimuli-Responsive Surface Modifications

Environmental sensitivity is incorporated into biogenic AgNP systems through stimuli-responsive surface engineering, providing a means of activating and controlling payload release via endogenous or exogenous input [37]. NPs can be triggered for action at known pathological microenvironments through surface modifications with pH-responsive polymers, redox-sensitive linkers, enzyme-cleavable bonds, thermo-responsive matrices, or photo-responsive moieties. This strategy enables therapeutic selectivity and decreases systemic toxicity by confining NPs bioactivity to target tissues. Therefore, stimuli-responsive biogenic AgNPs are an emerging dimension of smart nanomedicine and precision therapeutic engineering [130,131,132] (Table 4).

## 7. Precision Biomedical Applications

### 7.1. Targeted Anticancer Therapy

AgNPs from biogenic sources have exhibited immense potential as selective anticancer agents by conferring oxidative stress, causing mitochondrial dysfunction, and triggering DNA damage or apoptotic signaling to tumor cells [138]. Surface-engineered AgNPs modified with tumor-targeting ligands (e.g., folic acid, antibodies, peptides, or aptamers) can selectively localize in the tumor tissues by means of receptor-mediated endocytosis, as well as increased permeability and retention (EPR)-mediated mechanisms [139,140,141]. Internally, AgNPs can induce reactive oxygen species (ROS), cause mitochondrial membrane potential disruption, activate caspase cascades, and regulate several major oncogenic signaling pathways: the PI3K/Akt pathway, MAPK pathway, and p53-mediated apoptosis signaling pathway. Importantly, biogenic AgNPs also could be effectively applied for the delivery of chemotherapeutic agents in combination with synergistic chemo-nanotherapeutic strategies while exhibiting lower systemic toxicity. Despite promising preclinical findings, most anticancer applications of biogenic AgNPs remain at an early experimental stage. Challenges associated with systemic toxicity, biodistribution, immune interactions, long-term safety, and regulatory approval continue to limit clinical translation. Therefore, extensive in vivo validation and standardized toxicological assessment remain necessary before therapeutic implementation in precision oncology [142].

### 7.2. Antimicrobial and Anti-Biofilm Applications

One of the best-studied biomedical applications of AgNPs is their broad-spectrum antimicrobial activity against bacteria, fungi, viruses, and multidrug-resistant pathogens [143]. The first is biogenic AgNPs antimicrobial mechanisms acting via multiple simultaneous steps, i.e., membrane disruption, generation of ROS in cells, protein denaturation, and/or DNA damage or interference with microbial respiratory pathways [144]. Engineered metal NPs breach microbial biofilms and disrupt biofilm maturation by disintegrating extracellular polymeric matrices, as well as quorum-sensing-regulated biofilm maturation owing to their nanoscale dimensions [145]. Self-natured bioactive capping layers on the surface of biosynthesized AgNPs could also enhance their antimicrobial action by additive and/or synergistic interactions with different phytochemicals or extracts produced by microbial metabolites [146,147,148]. Among current biomedical applications, antimicrobial coatings, wound dressings, and topical anti-biofilm materials represent some of the most clinically advanced and translationally feasible uses of biogenic AgNPs.

### 7.3. Wound Healing and Tissue Engineering

Considering their unique combination of antimicrobial, anti-inflammatory, and pro-regenerative properties, biogenic AgNPs are becoming an invaluable component in wound dressings, scaffolds, hydrogels, and regenerative biomaterials [149]. AgNP-embedded wound dressings provide optimal microenvironments for healing by preventing microbial colonization and downregulation of excessive inflammatory responses [150]. Moreover, when silver ions are released in a controlled manner, they can lead to fibroblast migration as well as collagen deposition, angiogenesis, and re-epithelialization (hence promotes wound healing) [151,152]. The AgNP-functionalized scaffolds provide the next-generation biomaterials with desirable antibacterial properties and improved regenerative potential for skin, bone, and soft tissue engineering applications [153,154,155].

### 7.4. Drug Delivery Platforms

Biogenic AgNPs have also been used as helpful nanocarriers for small molecules, proteins, nucleic acids, and phytotherapeutics [156,157]. Their high surface-to-volume ratio and tailored surface chemistry enable efficient payload adsorption, encapsulation, or conjugation. Therapeutic agents can also be released from surface-functionalized polymers by using different stimuli-responsive linkers to enable controlled and site-specific release [135]. AgNP-based delivery systems of this sort might potentially improve pharmacokinetics and increase cellular uptake, tackle multidrug resistance, and ease off-target toxicity of therapeutics loaded therein [158,159].

### 7.5. Imaging and Theranostic Applications

**Figure 4:**
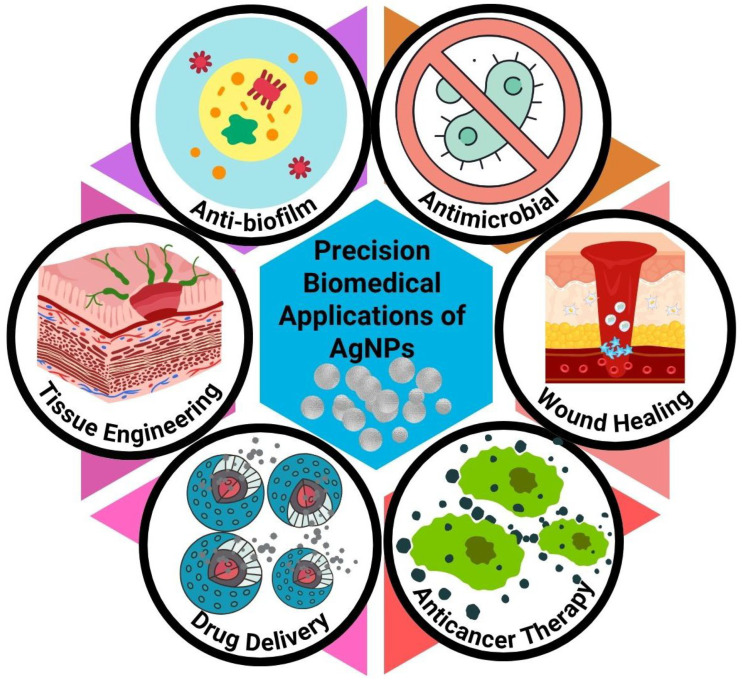
Schematic of key biomedical applications of AgNPs, including antimicrobial, anti-biofilm, anticancer, wound healing, tissue engineering, and drug delivery functions.

Due to the special optical and plasmonic characteristics, AgNPs are an attractive material for diagnostic imaging and theranostic applications [160]. Due to their LSPR properties, it facilitates strong optical scattering and absorption phenomena that allow applications such as surface-enhanced Raman spectroscopy, optical biosensing, photoacoustic imaging, and fluorescence enhancement [161,162]. Multifunctional biogenic AgNPs combining imaging capability with therapeutic responsiveness show promising potential as future theranostic platforms; however, most systems remain in preclinical investigation and require substantial validation regarding safety, targeting efficiency, and long-term clinical applicability (Figure 4) [136,137].

## 8. Nano–Bio Interactions and Safety Considerations

### 8.1. Cellular Uptake and Internalization

**Figure 5:**
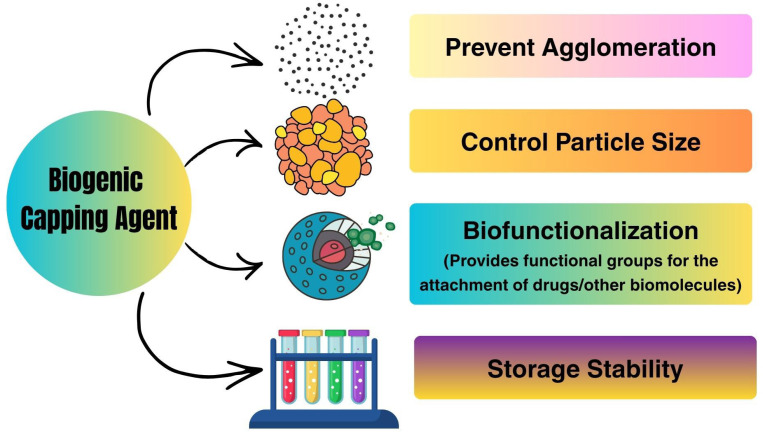
Role of biogenic capping agents in stabilizing AgNPs, highlighting their functions in preventing agglomeration, controlling particle size, enabling biofunctionalization for drug/biomolecule attachment, and enhancing storage stability.

The interaction of biogenic AgNPs with cellular membranes and their internalization routes is crucial for the biological performance of these ions [163]. Endocytic mechanisms for cellular uptake of AgNPs include clathrin-mediated endocytosis, caveolae-dependent uptake, macro pinocytosis or phagocytosis, which are determined by NPs size, morphology, surface charge, and functionalization [164]. In medicine, as a rule of thumb, smaller NPs have a higher internalization efficiency because they are more membrane-permeable and/or interact well with receptors on the membranes. Surface-coupled biological capping agents may also be involved in fine-tuning recognition by receptors and modifying intracellular trafficking pathways. After uptake, AgNPs are predominantly trafficked to endosomes and lysosomes, which could stimulate partial dissolution in acidic intracellular environments, ultimately resulting in the release of bioactive Ag^+^ ions [165,166] (Figure 5).

### 8.2. ROS Generation and Molecular Toxicity

Generation of reactive oxygen species has been identified as one of the principal mechanisms responsible for their biological activity and cytotoxicity [167]. Mitochondrial dysfunction, disruption of electron transport chains (ETCs), Fenton-like redox reactions, and depletion of endogenous antioxidant defenses can contribute to oxidative stress by intracellular AgNPs and released silver ions [168,169]. Increased levels of ROS induce lipid peroxidation, protein oxidation, breaks in DNA strands, and mitochondrial depolarization, as well as stress-responsive signaling pathways. Optimizing the dose is required, as excessive oxidative stress can indirectly contribute to unwanted cytotoxicity of healthy tissues; while therapeutic applications by AgNPs are explained mainly through controlled ROS generation coupled with stronger anticancer and antimicrobial effects. Smaller AgNPs generally show higher cellular uptake and oxidative stress due to increased surface area and Ag^+^ ion release. Excessive ROS generation may induce inflammation, mitochondrial dysfunction, DNA damage, and apoptosis in non-target tissues [170,171].

### 8.3. Immunomodulatory Effects

Biogenic AgNPs have a complex immunomodulatory effect determined by the physicochemical properties of genetic material, dose, and biological context [172]. Other NPs may activate innate immune pathways by a combination of macrophage, dendritic cells, neutrophils, and complement proteins. Depending on formulation parameters, AgNPs can elicit pro-inflammatory cytokine release, inflammasome activation, and oxidative immune responses or conversely suppress uncontrolled inflammation via antioxidant or anti-inflammatory capping biomolecules [173]. The biosynthetic nature of the corona is crucial for immune recognition and inflammation potential. It is hence important to focus on rational surface engineering, which minimizes unwanted immunogenicity and exploits favorable immunomodulatory functions [174,175,176].

### 8.4. Biodistribution and Pharmacokinetics

The biodistribution and pharmacokinetic behavior of biogenic AgNPs after systemic administration are affected by size, surface charge, hydrophobicity, aggregation state, and bio-corona composition [177]. AgNPs are largely sequestered by the mononuclear phagocyte system, with accumulation mainly in the liver, spleen, lungs, and kidneys. Surface functionalization approaches such as PEGylation and biomimetic coating achieve prolonged circulation time, which reduces quasi-rapid opsonization-mediated clearance [178]. No other nanoscale aspect of AgNPs can influence the kinetics of degradation and release profiles of silver ions, which affect tissue persistence and systemic exposure. However, complete pharmacokinetic profiling is still critical for the clinical utility of AgNP-based therapies. Systemically administered AgNPs may accumulate in the liver, spleen, kidneys, and lungs, raising concerns regarding chronic toxicity and delayed clearance. In contrast, localized applications such as wound dressings and antimicrobial coatings generally show lower systemic exposure and better translational feasibility [14].

### 8.5. Long-Term Biocompatibility Concerns

**Figure 6:**
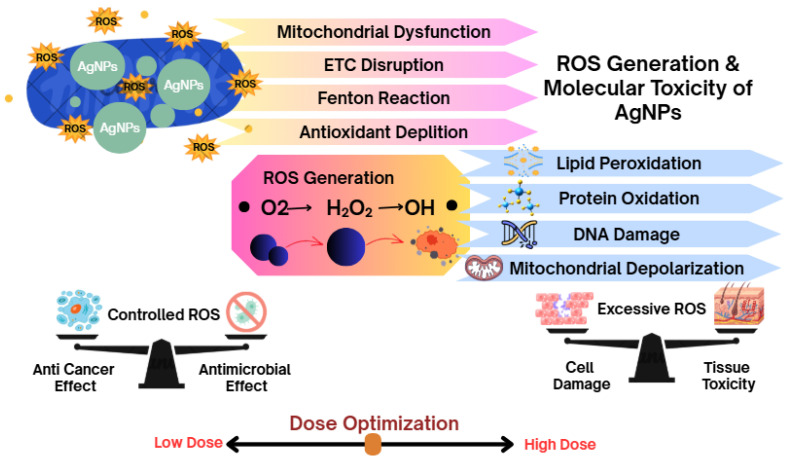
AgNPs induce ROS generation via mitochondrial dysfunction, ETC disruption, and Fenton-like reactions, leading to lipid, protein, and DNA damage. Controlled ROS promotes anticancer and antimicrobial effects, while excessive ROS causes cell and tissue toxicity, emphasizing the need for dose optimization.

While this offers a potentially useful therapeutic application, doubts persist as to the long-term safety of AgNPs exposure following repetitive or chronic administration. Adverse effects such as accumulation in lungs, chronic oxidative stress, mitochondrial dysfunction, genotoxicity, nephrotoxicity, hepatotoxicity, and reproductive neurotoxicity have been identified as potential hazards [179]. Long-term exposure can also disturb the host microbiota and interfere with physiological metal homeostasis. While biogenic synthesis and natural surface capping can enhance biocompatibility compared with chemically synthesized AgNPs, extensive persistence in vivo studies and standardized toxicological assessment paradigms are warranted to determine safety for clinical applications (Figure 6 and Table 5 and Table 6). Standardized long-term toxicological evaluation remains essential for safe clinical translation of biogenic AgNPs. Microbial-mediated AgNPs biosynthesis may also raise concerns regarding potential co-selection of metal and antibiotic resistance mechanisms under prolonged silver exposure. Certain microbial systems can activate metal-efflux pathways, stress-response systems, and resistance-associated genes that may contribute to reduced antimicrobial susceptibility. Therefore, careful biosafety evaluation and controlled therapeutic application remain important for long-term clinical translation [180,181].

## 9. Conclusions

Synthesis of biogenic AgNPs has become a sustainable and biologically favorable alternative to traditional physicochemical fabrication methods, providing eco-friendly production routes along with intrinsically biofunctionalized nanostructures that offer enormous biomedical potential. A broader scope of applications in AgNP biosynthesis and expanded biocompatibility and therapeutic incorporation of biological nanofactories, including plants, microorganisms, algae, fungi, and enzyme-directed systems. Nonetheless, most traditional green synthesis methods are still limited in mechanistic control, reproducibility, large batch variations, and scalability, thus hampering their translational potential. Key advancements in areas such as metabolic engineering, synthetic biology, microfluidic-assisted synthesis, artificial intelligence-aided optimization and process control, and continuous-flow biomanufacturing are reshaping this space by allowing precision biofabrication of AgNPs that feature greater uniformity, scalability, and functional tunability over current methods. Recent advances in surface engineering and functionalization have expanded the potential biomedical applications of biogenic AgNPs, particularly in antimicrobial materials, wound healing systems, and emerging targeted therapeutic platforms, although many advanced nanomedicine applications still require extensive preclinical and clinical validation. Future clinical translation of biogenic AgNPs will depend on standardized regulatory frameworks, GMP-compliant manufacturing, and rigorous quality control strategies. Substantial reduction in the amount of AgNPs in drug formulations and their effective utilization are anticipated by forming nanocompoistes based on biogenic AgNPs and biogenic heteroatom doped carbon nanodots and this new avenue need to be pursued actively.

## Figures and Tables

**Table 1:** Biological nanofactories for advanced AgNPs biosynthesis: mechanisms, functional attributes, and biomedical relevance.

Biological Platform	Representative Species/Examples	Principal Reducing/Stabilizing Components	Mechanism of AgNP Formation	Limitations	Representative Biomedical Relevance	References
Plant-Derived Systems	*Azadirachta indica*, *Camellia sinensis*, *Moringa oleifera*, *Aloe vera*	Polyphenols, flavonoids, terpenoids, alkaloids, reducing sugars, proteins	Phytochemical-mediated reduction of Ag^+^ followed by biomolecular capping/stabilization	Batch variability, extract heterogeneity, and poor standardization	Antimicrobial, antioxidant, anticancer, and wound healing	[73,74]
Bacterial Nanofactories	*Bacillus subtilis*, *Pseudomonas aeruginosa*, *Escherichia coli*, *Lactobacillus* spp.	Nitrate reductase, NADH-dependent reductases, exopolysaccharides, peptides	Intracellular/extracellular enzymatic reduction of Ag^+^ ions	Endotoxin contamination, biosafety concerns, and purification complexity	Drug delivery, antimicrobial coatings, biosensing	[75,76]
Fungal Nanofactories	*Fusarium oxysporum*, *Aspergillus niger*, *Penicillium* spp., *Candida albicans*	Nitrate reductase, laccase, quinone reductase, extracellular proteins, polysaccharides	Enzyme-mediated extracellular/intracellular reduction with protein-assisted stabilization	Longer cultivation time, contamination risk, variable metabolite secretion	Anticancer therapeutics, antimicrobial formulations	[77]
Algal Platforms	*Sargassum* spp., *Ulva lactuca*, *Chlorella vulgaris*, *Gracilaria* spp.	Sulfated polysaccharides, pigments, proteins, phenolics, antioxidants	Redox-active metabolite-mediated reduction and polysaccharide stabilization	Seasonal variability, biomass processing complexity	Anticancer, immunomodulatory, antimicrobial	[65]
Cyanobacterial Systems	*Spirulina platensis*, *Anabaena* spp., *Nostoc* spp.	Phycobiliproteins, chlorophyll derivatives, and extracellular polymeric substances	Pigment/protein-mediated reduction with extracellular stabilization	Limited industrial standardization, strain dependence	Biosensors, antimicrobial coatings	[64,65]
Enzyme-Directed/Cell-Free Systems	Purified nitrate reductase, laccase, glucose oxidase, isolated peptides	Purified reductases, oxidoreductases, peptides, isolated proteins/polysaccharides	Direct catalytic reduction under controlled conditions	High purification cost, enzyme instability, and limited scalability	Precision nanomedicine, targeted therapeutics	[70]

**Table 2:** Molecular mechanisms governing biogenic AgNPs formation.

Mechanistic Stage	Key Process	Major Biomolecules/Factors Involved	Functional Outcome	References
Ag^+^ Reduction	Conversion of silver ions to elemental silver (Ag^0^)	Polyphenols, flavonoids, terpenoids, NADH/NADPH, reductase enzymes	Initiation of NPs synthesis	[90]
Enzymatic Catalysis	Electron transfer-mediated metal reduction	Nitrate reductase, laccase, quinone reductase, and dehydrogenases	Accelerated Ag^+^ bioreduction	[76,77]
Nucleation	Aggregation of Ag^0^ atoms into stable nuclei	Supersaturated Ag^0^ atoms, redox-active biomolecules	Formation of primary NPs seeds	[81]
Growth Phase	Enlargement of nuclei via Ag^0^ deposition	Silver precursor concentration, pH, temperature, and biomolecule concentration	Determines size and morphology	[81]
Facet-Specific Growth Modulation	Selective adsorption on crystal faces	Proteins, peptides, polysaccharides, phenolics	Controls anisotropy/shape	[91]
Natural Capping	Surface coating of nascent NPs	Proteins, polysaccharides, polyphenols, lipids	Stabilization and anti-aggregation	[84]
Colloidal Stabilization	Electrostatic/steric repulsion between particles	Charged functional groups (–OH, –COOH, –NH_2_, –SH)	Enhanced dispersion stability	[5]
Bio-Corona Formation	Secondary adsorption of biomolecules in the biological milieu	Serum proteins, lipids, metabolites, nucleic acids	Defines biological identity and cellular interactions	[92]

**Table 3:** Advanced bioengineering strategies for controlled biogenic AgNPs fabrication.

Bioengineering Strategy	Core Principle	Specific Engineering Approaches	Impact on AgNP Synthesis	Major Advantages	Current Limitations/Challenges	Future Translational Potential	References
Metabolic Engineering of Microbial Nano Factories	Rational modification of microbial metabolic pathways to enhance reductive biosynthesis	Overexpression of reductase genes; enhancement of NADH/NADPH regeneration; modulation of glutathione pathways;	Increases Ag^+^ reduction efficiency, NPs yield, and physicochemical uniformity	Improved biosynthetic productivity, moderate reproducibility, and enhanced silver tolerance with potential scalability	Genetic instability, moderate scalability challenges, and strain-dependent reproducibility limitations	Promising for scalable industrial biosynthesis, but still at an early translational stage	[56]
Synthetic Biology Approaches	Construction of programmable genetic circuits for regulated NPs production	synthetic gene circuits; quorum sensing modules; feedback regulation loops; heterologous reductase/pathway	Enables temporal and conditional control of NPs formation	High precision and reproducibility under controlled systems with programmable nanoparticle synthesis	Technical complexity, regulatory concerns, and limited large-scale translational validation	Smart living nanofactories for on-demand nanomaterial synthesis	[90]
Microfluidic-Assisted Biogenic Synthesis	Microscale continuous-flow synthesis with precise control of reaction environment	Lab-on-chip reactors; laminar flow mixers; droplet microfluidics; segmented flow reactors; gradient generators	Improves nucleation uniformity and reduces polydispersity	High reproducibility, precise process control, and improved nanoparticle uniformity	Device fouling, scalability limitations, and high fabrication cost	Standardized continuous-flow precision nanomanufacturing	[100]
AI/ML-Guided Process Optimization	Computational prediction and optimization of synthesis parameters using data-driven models	Artificial neural networks; support vector machines; Bayesian optimization; predictive modeling	Predicts optimal synthesis conditions for desired AgNP characteristics	Accelerates optimization and improves reproducibility through predictive process control	Require large datasets; model overfitting risk; limited biological datasets	Autonomous self-optimizing NPs production platforms	[104,105]
Continuous-Flow Biomanufacturing	Steady-state NPs production in integrated flow systems	Continuous stirred tank reactors; tubular reactors; perfusion bioreactors; inline monitoring systems	Enables scalable and reproducible AgNP manufacturing	High scalability, improved reproducibility, and consistent nanoparticle quality	Purification complexity, sterility assurance, and GMP-compliance challenges	GMP-compatible industrial production of biogenic AgNPs	[100]
Bioreactor-Integrated Biosynthesis	Controlled cultivation of biological nanofactories in bioprocess systems	Fermentation optimization: pH/DO-controlled bioreactors; fed-batch cultivation; perfusion systems	Enhances biomass productivity and biosynthetic consistency	Scalable biological production; improved environmental control	High operational cost; contamination risk; process optimization needed	Large-scale fermentation-based AgNP manufacturing	[116]
Process Analytical Technology (PAT) Integration	Real-time monitoring and control of synthesis parameters	Inline UV-Vis spectroscopy; DLS monitoring; Raman spectroscopy; feedback-controlled automation	Maintains synthesis consistency and product quality	Quality assurance, process standardization, and reduced batch failure	High instrumentation cost; analytical integration challenges	Regulatory-compliant smart manufacturing systems	[115,116]

**Table 4:** Functionalization and surface engineering strategies for biogenic AgNPs.

Surface Engineering Strategy	Representative Modifications	Primary Purpose	Functional Outcome/Biomedical Benefit	References
Ligand Functionalization	PEG, folic acid, peptides, thiol ligands, targeting molecules	Improve stability and targeting	Enhanced circulation, receptor-mediated uptake, and reduced aggregation	[78]
Polymer Coating	Chitosan, Poly(lactic-co-glycolic acid), alginate, dextran, Polyvinyl alcohol	Surface protection and controlled release	Improved biocompatibility, sustained drug release, reduced toxicity	[133]
Hydrogel Encapsulation	Gelatin, alginate, collagen, composite hydrogels	Matrix embedding for localized delivery	Wound dressing, tissue regeneration, prolonged release	[120]
Antibody Conjugation	Monoclonal antibodies, receptor-specific antibodies	Targeted recognition of biomarkers	Selective binding and targeted therapy/diagnostics	[134]
Aptamer Functionalization	DNA/RNA aptamers	Molecular-specific targeting	High-affinity biosensing and targeted delivery	[128]
Drug Loading/Co-Delivery	Anticancer drugs, antibiotics, phytochemicals	Multifunctional therapeutic delivery	Synergistic therapeutic efficacy	[135]
pH-Responsive Modification	Acid-labile polymers/linkers	Tumor/infection-triggered activation	Site-specific release in acidic microenvironment	[81]
Redox/Enzyme Responsive Coating	Disulfide linkers, enzyme-cleavable shells	Triggered intracellular/pathological release	Enhanced therapeutic selectivity	[78]
Photo/Thermo-Responsive Functionalization	Photothermal dyes, thermo-sensitive polymers	External stimulus-mediated activation	Controlled release and theranostic applications	[136,137]

**Table 5:** Nano–Bio Interactions and Safety Considerations of Biogenic AgNPs.

Safety Aspect	Influencing Key Factors	Biological/Physiological Outcome	Potential Concern	References
Cellular Uptake	Size, shape, surface charge, ligand/capping layer	Endocytosis and intracellular trafficking	Excessive intracellular accumulation	[78]
ROS Generation	Ag^+^ release, mitochondrial interaction, redox activity	Oxidative stress induction	Damage to healthy cells	[167]
Molecular Toxicity	Dose, exposure time, and intracellular dissolution	DNA/protein/lipid damage, apoptosis	Cytotoxicity and genotoxicity	[169]
Immunomodulation	Surface chemistry, corona composition, dose	Cytokine modulation, immune activation/suppression	Inflammation or immunogenicity	[177]
Biodistribution	Particle size, PEGylation, hydrophobicity	Organ accumulation and systemic circulation	Off-target tissue deposition	[177]
Pharmacokinetics	Surface coating, aggregation state, degradation rate	Clearance and circulation half-life	Rapid clearance or prolonged retention	[177]
Long-Term Biocompatibility	Chronic exposure, repeated dosing	Organ toxicity, microbiota disturbance	Hepato/nephro/neurotoxicity	[179]
Bio-Corona Formation	Biological fluid composition, surface properties	Alters biological identity and uptake	Unpredictable in vivo behavior	[93,182]

**Table 6:** Major toxicological considerations associated with AgNPs.

Toxicity Parameter	Major Concern	Higher Risk	Lower Risk	References
Ag^+^ ion release	Oxidative stress	Systemic exposure	Local coatings	[181]
ROS generation	DNA and cell damage	High-dose exposure	Controlled release	[167]
Cytotoxicity	Cell membrane damage	Small AgNPs	Surface-coated AgNPs	[167]
Genotoxicity	DNA strand breaks	Chronic exposure	Short-term exposure	[172]
Inflammation	Cytokine activation	Repeated dosing	Biocompatible coatings	[167]
Biodistribution	Organ accumulation	Intravenous delivery	Topical application	[183]
Long-term toxicity	Tissue persistence	Systemic circulation	Localized treatment	[78]

## Data Availability

The original contributions presented in the study are included in the article, further inquiries can be directed to the corresponding authors.

## References

[1] Dayana B.M., Venkatesan R., Joseph Prakash J.T., Saravanan P., Nivetha M.S., Murali A., Vetcher A.A., Settu M., Kim S.-C. (2026). Biosynthesis of Silver Nanoparticles via Melaleuca Alternifolia Leaf Extract for Antibacterial, Antifungal, Antioxidant and Anticancer Activity. Sci. Rep..

[2] Kazi R.N.A., Hasani I.W., Khafaga D.S.R., Kabba S., Farhan M., Aatif M., Muteeb G., Fahim Y.A. (2025). Nanomedicine: The Effective Role of Nanomaterials in Healthcare from Diagnosis to Therapy. Pharmaceutics.

[3] Lakshmikanthan M., Muthu S., Krishnan K., Altemimi A.B., Abedelmaksoud T.G. (2026). Poly(d,l-Lactide-Co-Glycolide) (PLGA) Nanoparticles in Oncological Therapeutics: Mechanisms of Targeted Delivery and Encapsulation Strategies for Chemotherapeutics, Gene Modulators, and Phytochemicals. Nanomedicine.

[4] More P.R., Pandit S., De Filippis A., Franci G., Mijakovic I., Galdiero M. (2023). Silver Nanoparticles: Bactericidal and Mechanistic Approach against Drug Resistant Pathogens. Microorganisms.

[5] Nguyen N.P.U., Dang N.T., Doan L., Nguyen T.T.H. (2023). Synthesis of Silver Nanoparticles: From Conventional to ‘Modern’ Methods—A Review. Processes.

[6] Afonso I.S., Cardoso B., Nobrega G., Minas G., Ribeiro J.E., Lima R.A. (2024). Green Synthesis of Nanoparticles from Olive Oil Waste for Environmental and Health Applications: A Review. J. Environ. Chem. Eng..

[7] Calderon Moreno J.M., Chelu M., Popa M. (2026). Eco-Friendly Bioinspired Synthesis and Environmental Applications of Zinc Oxide Nanoparticles Mediated by Natural Polysaccharide Gums: A Sustainable Approach to Nanomaterials Fabrication. Nanomaterials.

[8] Sivalingam A.M. (2026). Green Synthesis and Characterization of Silver Nanoparticles (AgNPs) Using Bacopa Monnieri Leaf Extract Photoluminescence (PL) Profiling and Applications of Antioxidant, Antimicrobial Activity. Inorg. Chem. Commun..

[9] Dhaka A., Chand Mali S., Sharma S., Trivedi R. (2023). A review on biological synthesis of silver nanoparticles and their potential applications. Results Chem..

[10] Afzal S., Fatima M., Mishra M., Shukla N., Singh N.K., Sarraf M., Rastogi A. (2026). Insights into Progress in Biogenic Synthesis of Nanomaterials: A Review. Plant Nano Biol..

[11] Petrovic S., Bita B., Barbinta-Patrascu M.E. (2024). Nanoformulations in Pharmaceutical and Biomedical Applications: Green Perspectives. Int. J. Mol. Sci..

[12] Kamyab H., Khalili E., Khademi T., Yuzir A., Taheri M.M., Rajendran S., Peñaherrera-Pazmiño A.B. (2026). Advances in Green Synthesis of Nanoparticles for Biomedical Applications: Antimicrobial, Antiviral, and Cancer Therapies. Mater. Today Sustain..

[13] Ayyankalai N.K., Sundararaj P., Baskar M.G., Duraisamy N., Muthu S., Lakshmikanthan M., Abdi G. (2025). Green Production of Silver Nanoparticles from Cassia Occidentalis and Alternanthera Pungens and Evaluation of Their Nematicidal Activity against Meloidogyne Javanica. Sci. Rep..

[14] Rai M., Ingle A.P., Trzcińska-Wencel J., Wypij M., Bonde S., Yadav A., Kratošová G., Golińska P. (2021). Biogenic Silver Nanoparticles: What We Know and What Do We Need to Know?. Nanomaterials.

[15] Madhusudanan M., Mijakovic I., Singh P. (2025). Biogenic vs. Chemical AgNPs: A Comparison of Antimicrobial Potency and Stability. Int. J. Mol. Sci..

[16] Ntolia A., Chatzigiannakou T., Michailidis N., Aggeli A. (2025). A Comprehensive Physicochemical Characterization of Silver Nanoparticles as a Prerequisite for Their Successful Biomedical Applications. Inorganics.

[17] Gimondi S., Ferreira H., Reis R.L., Neves N.M. (2023). Microfluidic Devices: A Tool for Nanoparticle Synthesis and Performance Evaluation. ACS Nano.

[18] Foldi J., Connolly J.A., Takano E., Breitling R. (2024). Synthetic Biology of Natural Products Engineering: Recent Advances Across the Discover-Design-Build-Test-Learn Cycle. ACS Synth. Biol..

[19] Sharma S., Sinha P., Mohanty S., Khasa Y.P. (2025). Emerging Metabolic Engineering and Synthetic Biology Strategies in the Development of Lignocellulosic Biomass-Based Biorefineries. Lignocellulosic Biomass and Enzymes: Fundamentals, Emerging Technologies and Applications.

[20] Shahzadi S., Fatima S., Ul Ain Q., Shafiq Z., Janjua M.R.S.A. (2025). A Review on Green Synthesis of Silver Nanoparticles (SNPs) Using Plant Extracts: A Multifaceted Approach in Photocatalysis, Environmental Remediation, and Biomedicine. RSC Adv..

[21] Zahoor M., Nazir N., Iftikhar M., Naz S., Zekker I., Burlakovs J., Uddin F., Kamran A.W., Kallistova A., Pimenov N. (2021). A Review on Silver Nanoparticles: Classification, Various Methods of Synthesis, and Their Potential Roles in Biomedical Applications and Water Treatment. Water.

[22] Duman H., Eker F., Akdaşçi E., Witkowska A.M., Bechelany M., Karav S. (2024). Silver Nanoparticles: A Comprehensive Review of Synthesis Methods and Chemical and Physical Properties. Nanomaterials.

[23] Xiao H., Feng Y., Goundry W.R.F., Karlsson S. (2024). Organic Solvent Nanofiltration in Pharmaceutical Applications. Org. Process Res. Dev..

[24] Fernandes C., Jathar M., Sawant B.K.S., Warde T. (2023). Scale-Up of Nanoparticle Manufacturing Process. Pharmaceutical Process Engineering and Scale-Up Principles.

[25] Singh V., Sinha S., Verma J. (2026). Bioinformatics Models in Drug Delivery: Predicting Biomaterial-Biological Interactions for Targeted Therapies. Next Nanotechnol..

[26] Kolpek D.J., Kim J., Mohammed H., Gensel J.C., Park J. (2024). Physicochemical Property Effects on Immune Modulating Polymeric Nanoparticles: Potential Applications in Spinal Cord Injury. Int. J. Nanomed..

[27] Protik T.I., Ridoy M.N., Sazid M.G., Tanjim S., Supto J. (2026). Advances of Green Synthesized Nanomaterials in Different Industries. Mater. Proc..

[28] Singh H., Desimone M.F., Pandya S., Jasani S., George N., Adnan M., Aldarhami A., Bazaid A.S., Alderhami S.A. (2023). Revisiting the Green Synthesis of Nanoparticles: Uncovering Influences of Plant Extracts as Reducing Agents for Enhanced Synthesis Efficiency and Its Biomedical Applications. Int. J. Nanomed..

[29] Rathod S., Preetam S., Pandey C., Bera S.P. (2024). Exploring Synthesis and Applications of Green Nanoparticles and the Role of Nanotechnology in Wastewater Treatment. Biotechnol. Rep..

[30] Ilavenil K.K., Senthilkumar V., Kasthuri A. (2025). Green Synthesis of Metal Nanoparticles from Three Medicinal Plants: A Review of Environmental and Health Applications. Discov. Catal..

[31] Fahim M., Shahzaib A., Nishat N., Jahan A., Bhat T.A., Inam A. (2024). Green Synthesis of Silver Nanoparticles: A Comprehensive Review of Methods, Influencing Factors, and Applications. JCIS Open.

[32] Eker F., Akdaşçi E., Duman H., Bechelany M., Karav S. (2025). Green Synthesis of Silver Nanoparticles Using Plant Extracts: A Comprehensive Review of Physicochemical Properties and Multifunctional Applications. Int. J. Mol. Sci..

[33] Pandey P., Lakhanpal S., Bishoyi A.K., Jyothi S.R., Mishra S., Verma M., Singh A., Alam M.W., Rab S.O., Saeed M. (2025). Biosynthesis of Silver Nanoparticles from Plant Extracts: A Comprehensive Review Focused on Anticancer Therapy. Front. Pharmacol..

[34] Suárez Priede A., Gómez-Sánchez M., García-Cancela P., Bettmer J., Díez P. (2025). Deciphering the Formation of Biogenic Nanoparticles and Their Protein Corona: State-of-the-Art and Analytical Challenges. Anal. Bioanal. Chem..

[35] Hosny S., Gaber G.A., Ragab M.S., Ragheb M.A., Anter M., Mohamed L.Z. (2025). A Comprehensive Review of Silver Nanoparticles (AgNPs): Synthesis Strategies, Toxicity Concerns, Biomedical Applications, AI-Driven Advancements, Challenges, and Future Perspectives. Arab. J. Sci. Eng..

[36] Luzala M.M., Muanga C.K., Kyana J., Safari J.B., Zola E.N., Mbusa G.V., Nuapia Y.B., Liesse J.M.I., Nkanga C.I., Krause R.W.M. (2022). A Critical Review of the Antimicrobial and Antibiofilm Activities of Green-Synthesized Plant-Based Metallic Nanoparticles. Nanomaterials.

[37] Sati A., Ranade T.N., Mali S.N., Ahmad Yasin H.K., Pratap A. (2025). Silver Nanoparticles (AgNPs): Comprehensive Insights into Bio/Synthesis, Key Influencing Factors, Multifaceted Applications, and Toxicity—A 2024 Update. ACS Omega.

[38] Brooks S.M., Alper H.S. (2021). Applications, Challenges, and Needs for Employing Synthetic Biology beyond the Lab. Nat. Commun..

[39] Tang S., Tao J., Li Y. (2023). Challenges and Solutions for the Downstream Purification of Therapeutic Proteins. Antib. Ther..

[40] Ingram L.O., Jarboe L.R., Zhang X., Wang X., Moore J.C., Shanmugam K.T. (2010). Metabolic Engineering for Production of Biorenewable Fuels and Chemicals: Contributions of Synthetic Biology. J. Biomed. Biotechnol..

[41] Yan X., Liu X., Zhao C., Chen G.Q. (2023). Applications of Synthetic Biology in Medical and Pharmaceutical Fields. Signal Transduct. Target. Ther..

[42] Wang Q., Hu Z., Li Z., Liu T., Bian G. (2025). Exploring the Application and Prospects of Synthetic Biology in Engineered Living Materials. Adv. Mater..

[43] Hirschi S., Ward T.R., Meier W.P., Muller D.J., Fotiadis D. (2022). Synthetic Biology: Bottom-Up Assembly of Molecular Systems. Chem. Rev..

[44] Aal E Ali R.S., Meng J., Khan M.E.I., Jiang X. (2024). Machine Learning Advancements in Organic Synthesis: A Focused Exploration of Artificial Intelligence Applications in Chemistry. Artif. Intell. Chem..

[45] Lakshmikanthan M., Muthu S., Caleb J.T.D., Francis Y.M., Pulidindi I.N. (2025). Liver Cancer: Artificial Intelligence (AI)-Based Integrated Therapeutic Approaches. Bioengineering.

[46] Gricourt G., Meyer P., Duigou T., Faulon J.L. (2024). Artificial Intelligence Methods and Models for Retro-Biosynthesis: A Scoping Review. ACS Synth. Biol..

[47] Mehran M.J., Mohammadzadeh S., Bolideei M., Barzigar R., Haider K.H., Jadgal N., Bahrami Y. (2026). Artificial Intelligence in Drug Discovery: Integrative Advances From Data to Therapeutic Innovation. Drug Dev. Res..

[48] Shaikh W.A., Chakraborty S., Owens G., Islam R.U. (2021). A Review of the Phytochemical Mediated Synthesis of AgNP (Silver Nanoparticle): The Wonder Particle of the Past Decade. Appl. Nanosci..

[49] Chung I.M., Park I., Seung-Hyun K., Thiruvengadam M., Rajakumar G. (2016). Plant-Mediated Synthesis of Silver Nanoparticles: Their Characteristic Properties and Therapeutic Applications. Nanoscale Res. Lett..

[50] Guo Z., Chakraborty S., Monikh F.A., Varsou D.D., Chetwynd A.J., Afantitis A., Lynch I., Zhang P. (2021). Surface Functionalization of Graphene-Based Materials: Biological Behavior, Toxicology, and Safe-By-Design Aspects. Adv. Biol..

[51] Sapsford K.E., Algar W.R., Berti L., Gemmill K.B., Casey B.J., Oh E., Stewart M.H., Medintz I.L. (2013). Functionalizing Nanoparticles with Biological Molecules: Developing Chemistries That Facilitate Nanotechnology. Chem. Rev..

[52] Burlec A.F., Corciova A., Boev M., Batir-Marin D., Mircea C., Cioanca O., Danila G., Danila M., Bucur A.F., Hancianu M. (2023). Current Overview of Metal Nanoparticles’ Synthesis, Characterization, and Biomedical Applications, with a Focus on Silver and Gold Nanoparticles. Pharmaceuticals.

[53] Chatterjee A., Kwatra N., Abraham J. (2020). Nanoparticles Fabrication by Plant Extracts. Phytonanotechnology: Challenges and Prospects.

[54] Koul B., Poonia A.K., Yadav D., Jin J.O. (2021). Microbe-Mediated Biosynthesis of Nanoparticles: Applications and Future Prospects. Biomolecules.

[55] Wang X., Lee S.Y., Akter S., Huq M.A. (2022). Probiotic-Mediated Biosynthesis of Silver Nanoparticles and Their Antibacterial Applications against Pathogenic Strains of Escherichia Coli O157:H7. Polymers.

[56] Bamal D., Singh A., Chaudhary G., Kumar M., Singh M., Rani N., Mundlia P., Sehrawat A.R. (2021). Silver Nanoparticles Biosynthesis, Characterization, Antimicrobial Activities, Applications, Cytotoxicity and Safety Issues: An Updated Review. Nanomaterials.

[57] Mohammadi Dargah M., Pedram P., Cabrera-Barjas G., Delattre C., Nesic A., Santagata G., Cerruti P., Moeini A. (2024). Biomimetic Synthesis of Nanoparticles: A Comprehensive Review on Green Synthesis of Nanoparticles with a Focus on Prosopis Farcta Plant Extracts and Biomedical Applications. Adv. Colloid Interface Sci..

[58] Guilger-Casagrande M., de Lima R. (2019). Synthesis of Silver Nanoparticles Mediated by Fungi: A Review. Front. Bioeng. Biotechnol..

[59] Li G., He D., Qian Y., Guan B., Gao S., Cui Y., Yokoyama K., Wang L. (2011). Fungus-Mediated Green Synthesis of Silver Nanoparticles Using Aspergillus Terreus. Int. J. Mol. Sci..

[60] Lakshmikanthan M., Muthu S., Mohamed P.M.D., Kathiravan K. (2025). Molecular Characterization and Bioactive Potential of Sulfated Galactan Gssg-2 from Gracilaria Salicornia. SSRN.

[61] Othman A.M., Elsayed M.A., Al-Balakocy N.G., Hassan M.M., Elshafei A.M. (2019). Biosynthesis and Characterization of Silver Nanoparticles Induced by Fungal Proteins and Its Application in Different Biological Activities. J. Genet. Eng. Biotechnol..

[62] Malik M.A., Wani A.H., Bhat M.Y., Siddiqui S., Alamri S.A.M., Alrumman S.A. (2024). Fungal-Mediated Synthesis of Silver Nanoparticles: A Novel Strategy for Plant Disease Management. Front. Microbiol..

[63] Hamida R.S., Ali M.A., Redhwan A., Bin-Meferij M.M. (2020). Cyanobacteria—A Promising Platform in Green Nanotechnology: A Review on Nanoparticles Fabrication and Their Prospective Applications. Int. J. Nanomed..

[64] Chabili A., Minaoui F., Hakkoum Z., Douma M., Meddich A., Loudiki M. (2024). A Comprehensive Review of Microalgae and Cyanobacteria-Based Biostimulants for Agriculture Uses. Plants.

[65] Singh S., Kate B.N., Banecjee U.C. (2005). Bioactive Compounds from Cyanobacteria and Microalgae: An Overview. Crit. Rev. Biotechnol..

[66] Rojas V., Rivas L., Cárdenas C., Guzmán F. (2020). Cyanobacteria and Eukaryotic Microalgae as Emerging Sources of Antibacterial Peptides. Molecules.

[67] Bhardwaj A.K., Naraian R. (2021). Cyanobacteria as Biochemical Energy Source for the Synthesis of Inorganic Nanoparticles, Mechanism and Potential Applications: A Review. 3 Biotech..

[68] Hooe S.L., Smith A.D., Dean S.N., Breger J.C., Ellis G.A., Medintz I.L. (2024). Multienzymatic Cascades and Nanomaterial Scaffolding—A Potential Way Forward for the Efficient Biosynthesis of Novel Chemical Products. Adv. Mater..

[69] Rice A.J., Sword T.T., Chengan K., Mitchell D.A., Mouncey N.J., Moore S.J., Bailey C.B. (2025). Cell-Free Synthetic Biology for Natural Product Biosynthesis and Discovery. Chem. Soc. Rev..

[70] Lee S.J., Kim D.M. (2024). Cell-Free Synthesis: Expediting Biomanufacturing of Chemical and Biological Molecules. Molecules.

[71] Dudley Q.M., Karim A.S., Jewett M.C. (2014). Cell-Free Metabolic Engineering: Biomanufacturing beyond the Cell. Biotechnol. J..

[72] Nyabadza A., McCarthy É., Makhesana M., Heidarinassab S., Plouze A., Vazquez M., Brabazon D. (2023). A Review of Physical, Chemical and Biological Synthesis Methods of Bimetallic Nanoparticles and Applications in Sensing, Water Treatment, Biomedicine, Catalysis and Hydrogen Storage. Adv. Colloid Interface Sci..

[73] Shyamkiran Singh S. (2025). Green Synthesis of Nanoparticles Using Medicinal Plants: Mechanisms, Applications, and Future Prospects. Int. J. Multidiscip. Res..

[74] Villagrán Z., Anaya-Esparza L.M., Velázquez-Carriles C.A., Silva-Jara J.M., Ruvalcaba-Gómez J.M., Aurora-Vigo E.F., Rodríguez-Lafitte E., Rodríguez-Barajas N., Balderas-León I., Martínez-Esquivias F. (2024). Plant-Based Extracts as Reducing, Capping, and Stabilizing Agents for the Green Synthesis of Inorganic Nanoparticles. Resources.

[75] Zong R., Ruan H., Liu C., Fan S., Li J. (2023). Bacteria and Bacterial Components as Natural Bio-Nanocarriers for Drug and Gene Delivery Systems in Cancer Therapy. Pharmaceutics.

[76] Cheng Q., Zhu Y., Lv S., Shi J., Kuang M., Wang L., Ji X. (2025). Engineered Bacteria and Bacteria-Derived Nanomaterials for Cancer Therapy: Mechanisms, Designs and Advances. Bioact. Mater..

[77] Moghaddam A.B., Namvar F., Moniri M., Tahir P.M., Azizi S., Mohamad R. (2015). Nanoparticles Biosynthesized by Fungi and Yeast: A Review of Their Preparation, Properties, and Medical Applications. Molecules.

[78] Yadav M., Gaur N., Wahi N., Singh S., Kumar K., Amoozegar A., Sharma E. (2025). Phytochemical-Assisted Fabrication of Biogenic Silver Nanoparticles from Vitex negundo: Structural Features, Antibacterial Activity, and Cytotoxicity Evaluation. Colloids Interfaces.

[79] Mikhailova E.O. (2020). Silver Nanoparticles: Mechanism of Action and Probable Bio-Application. J. Funct. Biomater..

[80] Iravani S., Korbekandi H., Mirmohammadi S.V., Zolfaghari B. (2014). Synthesis of Silver Nanoparticles: Chemical, Physical and Biological Methods. Res. Pharm. Sci..

[81] Salih A.M., Al-Qurainy F., Khan S., Nadeem M., Tarroum M., Shaikhaldein H.O. (2022). Biogenic Silver Nanoparticles Improve Bioactive Compounds in Medicinal Plant Juniperus Procera In Vitro. Front. Plant Sci..

[82] Saha P., Mahiuddin M., Islam A.B.M.N., Ochiai B. (2021). Biogenic Synthesis and Catalytic Efficacy of Silver Nanoparticles Based on Peel Extracts of Citrus Macroptera Fruit. ACS Omega.

[83] Krauss I.R., Merlino A., Vergara A., Sica F. (2013). An Overview of Biological Macromolecule Crystallization. Int. J. Mol. Sci..

[84] Patakfalvi R., Papp S., Dékány I. (2007). The Kinetics of Homogeneous Nucleation of Silver Nanoparticles Stabilized by Polymers. J. Nanoparticle Res..

[85] Abbas R., Luo J., Qi X., Naz A., Khan I.A., Liu H., Yu S., Wei J. (2024). Silver Nanoparticles: Synthesis, Structure, Properties and Applications. Nanomaterials.

[86] Richards V.N., Rath N.P., Buhro W.E. (2010). Pathway from a Molecular Precursor to Silver Nanoparticles: The Prominent Role of Aggregative Growth. Chem. Mater..

[87] Sidhu A.K., Verma N., Kaushal P. (2022). Role of Biogenic Capping Agents in the Synthesis of Metallic Nanoparticles and Evaluation of Their Therapeutic Potential. Front. Nanotechnol..

[88] Bulgarini A., Lampis S., Turner R.J., Vallini G. (2020). Biomolecular Composition of Capping Layer and Stability of Biogenic Selenium Nanoparticles Synthesized by Five Bacterial Species. Microb. Biotechnol..

[89] Naganthran A., Verasoundarapandian G., Khalid F.E., Masarudin M.J., Zulkharnain A., Nawawi N.M., Karim M., Abdullah C.A.C., Ahmad S.A. (2022). Synthesis, Characterization and Biomedical Application of Silver Nanoparticles. Materials.

[90] Raj S., Trivedi R., Soni V. (2021). Biogenic Synthesis of Silver Nanoparticles, Characterization and Their Applications—A Review. Surfaces.

[91] Li Y., Lin H., Zhou W., Sun L., Samanta D., Mirkin C.A. (2021). Corner-, Edge-, and Facet-Controlled Growth of Nanocrystals. Sci. Adv..

[92] Shannahan J.H., Podila R., Brown J.M. (2015). A Hyperspectral and Toxicological Analysis of Protein Corona Impact on Silver Nanoparticle Properties, Intracellular Modifications, and Macrophage Activation. Int. J. Nanomed..

[93] Shannahan J.H., Lai X., Ke P.C., Podila R., Brown J.M., Witzmann F.A. (2013). Silver Nanoparticle Protein Corona Composition in Cell Culture Media. PLoS ONE.

[94] Akhter M.H., Khalilullah H., Gupta M., Alfaleh M.A., Alhakamy N.A., Riadi Y., Md S. (2021). Impact of Protein Corona on the Biological Identity of Nanomedicine: Understanding the Fate of Nanomaterials in the Biological Milieu. Biomedicines.

[95] Yaraki M.T., Zahed Nasab S., Zare I., Dahri M., Moein Sadeghi M., Koohi M., Tan Y.N. (2022). Biomimetic Metallic Nanostructures for Biomedical Applications, Catalysis, and Beyond. Ind. Eng. Chem. Res..

[96] Srujana T.L., Rao K.J., Korumilli T. (2025). Natural Biogenic Templates for Nanomaterial Synthesis: Advances, Applications, and Environmental Perspectives. ACS Biomater. Sci. Eng..

[97] Rodrigues A.S., Batista J.G.S., Rodrigues M.Á.V., Thipe V.C., Minarini L.A.R., Lopes P.S., Lugão A.B. (2024). Advances in Silver Nanoparticles: A Comprehensive Review on Their Potential as Antimicrobial Agents and Their Mechanisms of Action Elucidated by Proteomics. Front. Microbiol..

[98] Iravani S., Varma R.S. (2020). Bacteria in Heavy Metal Remediation and Nanoparticle Biosynthesis. ACS Sustain. Chem. Eng..

[99] Mukherjee S., Verma A., Kong L., Rengan A.K., Cahill D.M., Mukherjee S., Verma A., Kong L., Rengan A.K., Cahill D.M. (2024). Advancements in Green Nanoparticle Technology: Focusing on the Treatment of Clinical Phytopathogens. Biomolecules.

[100] Rabani M.S., Shrivastav M., Fayaz M., Pathak A., Khan M., Bashir S.U., Hamid I., Hussain H., Sharma J.K., Gupta M.K. (2026). Mechanistic Advances in Microbial Nanobiotechnology and Their Applications in Sustainable Agriculture, Environment and Biomedicine. Discov. Nano.

[101] Wang Y.H., Wei K.Y., Smolke C.D. (2013). Synthetic Biology: Advancing the Design of Diverse Genetic Systems. Annu. Rev. Chem. Biomol. Eng..

[102] Lee H.M., Vo P.N.L., Na D. (2018). Advancement of Metabolic Engineering Assisted by Synthetic Biology. Catalysts.

[103] Mehulkumar Joshi N., Gharia B., Mahavir B. (2024). Harnessing the Power of Life: Synthetic Biology for next-Generation Chemical Synthesis. J. Pharm. Res. Sci. Technol..

[104] Tuan D.A., Uyen P.V.N., Binh L.A., Nhan N.V.T., Hung L.T., Trinh N., Linh N.D., Masak J. (2024). Harnessing Quorum Sensing for Advanced Biotechnological Applications: Intra- and Inter-Species Communication for Synthetic Biology and Disease Control. Preprints.

[105] Ge C., Yu Z., Sheng H., Shen X., Sun X., Zhang Y., Yan Y., Wang J., Yuan Q. (2022). Redesigning Regulatory Components of Quorum-Sensing System for Diverse Metabolic Control. Nat. Commun..

[106] Nathanael K., Kovalchuk N.M., Simmons M.J.H. (2025). Comparison of Microfluidic Synthesis of Silver Nanoparticles in Flow and Drop Reactors at Low Dean Numbers. Micromachines.

[107] Khizar S., Zine N., Errachid A., Jaffrezic-Renault N., Elaissari A. (2022). Microfluidic-Based Nanoparticle Synthesis and Their Potential Applications. Electrophoresis.

[108] Langguth K.J., Maccagnano-Zachera S., Heinemann J. (2024). Microfluidic Production of Silver Nanoparticles Demonstrates Ability for on Demand Synthesis of a Wide Size Distribution of Particles. J. Nanoparticle Res..

[109] Agha A., Waheed W., Stiharu I., Nerguizian V., Destgeer G., Abu-Nada E., Alazzam A. (2023). A Review on Microfluidic-Assisted Nanoparticle Synthesis, and Their Applications Using Multiscale Simulation Methods. Discov. Nano.

[110] Furxhi I., Faccani L., Zanoni I., Brigliadori A., Vespignani M., Costa A.L. (2024). Design Rules Applied to Silver Nanoparticles Synthesis: A Practical Example of Machine Learning Application. Comput. Struct. Biotechnol. J..

[111] Dorsey P.J., Lau C.L., Chang T.C., Doerschuk P.C., D’Addio S.M. (2024). Review of Machine Learning for Lipid Nanoparticle Formulation and Process Development. J. Pharm. Sci..

[112] Vengateswaran H.T., Habeeb M., Ahmed R., You H.W., Kumbhar S.T., Chenchu Lakshmi K.N.V., Gorde P.L. (2026). Integrating Artificial Intelligence for Design, Optimization and Pharmacokinetic Prediction in Nanoparticle Based Drug Delivery. J. Drug Deliv. Sci. Technol..

[113] Othman Z.K., Ahmed M.M., Kasimieh O., Musa S.S., Branda F., Cue E.G., Ocampo J.M.A., Lucero Prisno D.E., Vimolmangkang S. (2025). Artificial Intelligence for Natural Product Drug Discovery and Development: Current Landscape, Applications, and Future Directions. Intell. Based Med..

[114] Vora L.K., Gholap A.D., Jetha K., Thakur R.R.S., Solanki H.K., Chavda V.P. (2023). Artificial Intelligence in Pharmaceutical Technology and Drug Delivery Design. Pharmaceutics.

[115] Cheng Y., Bi X., Xu Y., Liu Y., Li J., Du G., Lv X., Liu L. (2023). Artificial Intelligence Technologies in Bioprocess: Opportunities and Challenges. Bioresour. Technol..

[116] Ros H., Chan N., Cook M.T., Shorthouse D. (2026). Artificial Intelligence and Machine Learning Guided Optimization in Drug Delivery. Adv. Drug Deliv. Rev..

[117] Lin X.Z., Terepka A.D., Yang H. (2004). Synthesis of Silver Nanoparticles in a Continuous Flow Tubular Microreactor. Nano Lett..

[118] Yaghmaei M., Bourgonje C.R., Scaiano J.C. (2023). Facile Scale-Up of the Flow Synthesis of Silver Nanostructures Based on Norrish Type I Photoinitiators. Molecules.

[119] Costa C., Padrela L. (2025). Progress on Drug Nanoparticle Manufacturing: Exploring the Adaptability of Batch Bottom-up Approaches to Continuous Manufacturing. J. Drug Deliv. Sci. Technol..

[120] Pai S., Shetty K.V. (2025). Scaling up Green Synthesis of Silver Nanoparticles and Their Immobilization for Water Disinfection in a “Point of Use” Fixed Bed Unit. J. Water Process Eng..

[121] Jamieson P., Bannon R., Cronly D., Smyth M., Wharry S., Moody T.S., Lennon G., Baumann M. (2026). The Impact of Continuous Flow Technology and Collaboration between Academia and Industry. JACS Au.

[122] Srinivasan S., Gollapelli B., Soundarajan R.K., Sonawane S.H. (2025). A Process-Intensified Approach for Large-Scale Manufacturing of Silver Nanowires Using a Continuous Flow Reactor: Experiments and Application. Ind. Eng. Chem. Res..

[123] Dwivedi S.D., Singh D., Singh M.R. (2024). Engineering and Optimization of Biogenic Silver Nanoparticles: A Multimodal Approach to Glucocorticoid-Based Rheumatoid Arthritis Management. BioNanoScience.

[124] Laib I., Gheraissa N., Benaissa A., Benkhira L., Azzi M., Benaissa Y., Abdelaziz A.G., Tian F., Walsh M., Bechelany M. (2025). Tailoring Innovative Silver Nanoparticles for Modern Medicine: The Importance of Size and Shape Control and Functional Modifications. Mater. Today Bio.

[125] Nicolae-Maranciuc A., Chicea D. (2025). Polymeric Systems as Hydrogels and Membranes Containing Silver Nanoparticles for Biomedical and Food Applications: Recent Approaches and Perspectives. Gels.

[126] Muthu S., Altemimi A.B., Lakshmikanthan M., Krishnan K., ALKaisy Q.H., Awlqadr F.H., Hesarinejad M.A. (2025). Phycocolloids from Sargassum Microcystum: Immunomodulatory and Antioxidant Activities of Alginic Acid and Fucoidan. Food Hydrocoll. Health.

[127] Tabrizi E., Li B. (2025). Silver Integrated Hybrids and Nanocomposites for Next-Generation Biomedicine: Beyond Antimicrobial Coatings toward Smart Sense–Response–Heal Platforms. Mater. Today Bio.

[128] Fu Z., Xiang J. (2020). Aptamer-Functionalized Nanoparticles in Targeted Delivery and Cancer Therapy. Int. J. Mol. Sci..

[129] Ammar M.M., Ali R., Abd Elaziz N.A., Habib H., Abbas F.M., Yassin M.T., Maniah K., Abdelaziz R. (2025). Nanotechnology in Oncology: Advances in Biosynthesis, Drug Delivery, and Theranostics. Discov. Oncol..

[130] Chandrakala V., Aruna V., Angajala G. (2022). Review on Metal Nanoparticles as Nanocarriers: Current Challenges and Perspectives in Drug Delivery Systems. Emergent Mater..

[131] Parvin N., Aslam M., Joo S.W., Mandal T.K. (2025). Nano-Phytomedicine: Harnessing Plant-Derived Phytochemicals in Nanocarriers for Targeted Human Health Applications. Molecules.

[132] Firouzpour H., Najafi F., Mohammadgholi A., Alizadegan D., Mahmoudi Beram F., Akhtari N., Zoghi M., Ansar N., Tavakkoli Yaraki M. (2026). Nanoparticle-Based Drug Delivery Systems for Effective Cancer Treatment: Mechanisms and Applications. Next Nanotechnol..

[133] Lakshmikanthan M., Abdulla M., Krishnan K., Moovendhan M., Muthu S. (2026). Anthocyanin-Loaded Chitosan-Alginate Nanoparticles from Red Dragon Fruit Pulp for Targeted Therapy against HCT116 Colon Cancer Cells. Int. J. Biol. Macromol..

[134] Hamzah H. (2026). Targeted Therapy and Biomarker-Guided Applications of Ecofriendly Silver Nanoparticles in Precision Oncology. J. Pharm. Pharm. Sci..

[135] Khalil A.K.A., Teow Y.H., Takriff M.S., Ahmad A.L., Atieh M.A. (2025). Recent developments in stimuli-responsive polymer for emerging applications: A review. Results Eng..

[136] Caro C., Castillo P.M., Klippstein R., Pozo D., Zaderenko A.P. (2010). Silver Nanoparticles: Sensing and Imaging Applications. Silver Nanoparticles.

[137] Lee S.H., Jun B.H. (2019). Silver Nanoparticles: Synthesis and Application for Nanomedicine. Int. J. Mol. Sci..

[138] Kah G., Chandran R., Abrahamse H. (2023). Biogenic Silver Nanoparticles for Targeted Cancer Therapy and Enhancing Photodynamic Therapy. Cells.

[139] Khan M.S., Alomari A., Tabrez S., Hassan I., Wahab R., Bhat S.A., Alafaleq N.O., Altwaijry N., Shaik G.M., Zaidi S.K. (2021). Anticancer Potential of Biogenic Silver Nanoparticles: A Mechanistic Study. Pharmaceutics.

[140] Takáč P., Michalková R., Čižmáriková M., Bedlovičová Z., Balážová Ľ., Takáčová G. (2023). The Role of Silver Nanoparticles in the Diagnosis and Treatment of Cancer: Are There Any Perspectives for the Future?. Life.

[141] Ma J., Hai Y., Zheng K., Hu X., Ni K. (2025). Nanoparticle-based drug delivery systems in urologic oncology: From targeted therapy to precision theranostics. Mater. Today Bio.

[142] Yuan Y.G., Zhang S., Hwang J.Y., Kong I.K. (2018). Silver Nanoparticles Potentiates Cytotoxicity and Apoptotic Potential of Camptothecin in Human Cervical Cancer Cells. Oxid. Med. Cell. Longev..

[143] Khan M., Khan A.U., Bogdanchikova N., Garibo D. (2021). Antibacterial and Antifungal Studies of Biosynthesized Silver Nanoparticles against Plant Parasitic Nematode Meloidogyne Incognita, Plant Pathogens Ralstonia Solanacearum and Fusarium Oxysporum. Molecules.

[144] Okur E.E., Akdaşçi E., Eker F., Bechelany M., Karav S. (2026). Silver Nanoparticles as Anticancer Agents: Mechanisms Insight, Current Studies, and Limitations. Pharmaceuticals.

[145] Mohanta Y.K., Biswas K., Jena S.K., Hashem A., Abd_Allah E.F., Mohanta T.K. (2020). Anti-Biofilm and Antibacterial Activities of Silver Nanoparticles Synthesized by the Reducing Activity of Phytoconstituents Present in the Indian Medicinal Plants. Front. Microbiol..

[146] Khalifa H.O., Oreiby A., Mohammed T., Abdelhamid M.A.A., Sholkamy E.N., Hashem H., Fereig R.M. (2025). Silver Nanoparticles as Next-Generation Antimicrobial Agents: Mechanisms, Challenges, and Innovations against Multidrug-Resistant Bacteria. Front. Cell. Infect. Microbiol..

[147] Luceri A., Francese R., Lembo D., Ferraris M., Balagna C. (2023). Silver Nanoparticles: Review of Antiviral Properties, Mechanism of Action and Applications. Microorganisms.

[148] Elouardy K., Mouzaki M., Ahmoum H., Akhrouf A., Faik A., Mir Y. (2025). The Impact of PH on the Size of Biosynthesized Silver Nanoparticles: Evaluation of Antioxidant and Antibacterial Activities. Nano-Struct. Nano-Objects.

[149] Astaneh M.E., Fereydouni N. (2024). Silver Nanoparticles in 3D Printing: A New Frontier in Wound Healing. ACS Omega.

[150] Huang C., Dong L., Zhao B., Lu Y., Huang S., Yuan Z., Luo G., Xu Y., Qian W. (2022). Anti-inflammatory Hydrogel Dressings and Skin Wound Healing. Clin. Transl. Med..

[151] Banerjee D., Vydiam K., Vangala V., Mukherjee S. (2025). Advancement of Nanomaterials- and Biomaterials-Based Technologies for Wound Healing and Tissue Regenerative Applications. ACS Appl. Bio Mater..

[152] Alberts A., Tudorache D.I., Niculescu A.G., Grumezescu A.M. (2025). Advancements in Wound Dressing Materials: Highlighting Recent Progress in Hydrogels, Foams, and Antimicrobial Dressings. Gels.

[153] da Silva D.P., Silva A.d.S., da Silva J.V.M., Silva L.M.d.M., Neto J.J.d.S., Moreira I.F., Moreira R.T.d.F., Silva A.C.A. (2025). Emerging Trends in Wound Management Using Alginate-Based Dressings Functionalized with Metallic Nanoparticles: A Scoping Review. J. Explor. Res. Pharmacol..

[154] Zivari-Ghader T., Rashidi M.R., Mehrali M. (2024). Biological Macromolecule-Based Hydrogels with Antibacterial and Antioxidant Activities for Wound Dressing: A Review. Int. J. Biol. Macromol..

[155] Rybka M., Mazurek Ł., Konop M. (2022). Beneficial Effect of Wound Dressings Containing Silver and Silver Nanoparticles in Wound Healing—From Experimental Studies to Clinical Practice. Life.

[156] Abdel-Megeed R.M. (2024). Biogenic Nanoparticles as a Promising Drug Delivery System. Toxicol. Rep..

[157] Jiang X., Khan S., Dykes A., Stulz E., Zhang X. (2025). Biogenic Synthesis of Silver Nanoparticles and Their Diverse Biomedical Applications. Molecules.

[158] Yusuf A., Almotairy A.R.Z., Henidi H., Alshehri O.Y., Aldughaim M.S. (2023). Nanoparticles as Drug Delivery Systems: A Review of the Implication of Nanoparticles’ Physicochemical Properties on Responses in Biological Systems. Polymers.

[159] Harun-Ur-Rashid M., Foyez T., Krishna S.B.N., Poda S., Imran A. (2025). Bin Recent Advances of Silver Nanoparticle-Based Polymer Nanocomposites for Biomedical Applications. RSC Adv..

[160] Hang Y., Wang A., Wu N. (2024). Plasmonic Silver and Gold Nanoparticles: Shape- and Structure-Modulated Plasmonic Functionality for Point-of-Caring Sensing, Bio-Imaging and Medical Therapy. Chem. Soc. Rev..

[161] Schira R., Rabilloud F. (2019). Localized Surface Plasmon Resonance in Free Silver Nanoclusters Agn, n = 20–147. J. Phys. Chem. C.

[162] Melo R.M., Albuquerque G.M., Monte J.P., Pereira G.A.L., Pereira G. (2025). Recent Advances in the Application of Silver Nanoparticles for Enhancing Phototherapy Outcomes. Pharmaceuticals.

[163] Dutta R., Madan A. (2025). Gold and Silver Nanoparticles in Biomedical Applications: Cytotoxicity, Mechanism of Action, and Future Challenges. Drug Deliv. Lett..

[164] Sousa De Almeida M., Susnik E., Drasler B., Taladriz-Blanco P., Petri-Fink A., Rothen-Rutishauser B. (2021). Understanding Nanoparticle Endocytosis to Improve Targeting Strategies in Nanomedicine. Chem. Soc. Rev..

[165] Takáč P., Michalková R., Čižmáriková M., Bedlovičová Z., Balážová Ľ., Laca Megyesi Š., Mačeková Z., Takáčová G., Moreno-Borrallo A., Ruiz-Hernandez E. (2025). Do We Know Enough About the Safety Profile of Silver Nanoparticles in Oncology? A Focus on Novel Methods and Approaches. Int. J. Mol. Sci..

[166] Joseph T.M., Kar Mahapatra D., Esmaeili A., Piszczyk Ł., Hasanin M.S., Kattali M., Haponiuk J., Thomas S. (2023). Nanoparticles: Taking a Unique Position in Medicine. Nanomaterials.

[167] Akter S., Madhuvilakku R., Kar A.K., Nila I.S., Liu P., Inuzuka H., Wei W., Hong Y. (2026). Reactive Oxygen Species (ROS) in Cancer: From Mechanism to Therapeutic Implications. Signal Transduct. Target. Ther..

[168] Villalpando-Rodriguez G.E., Gibson S.B. (2021). Reactive Oxygen Species (ROS) Regulates Different Types of Cell Death by Acting as a Rheostat. Oxid. Med. Cell. Longev..

[169] Ullah I., Khalil A.T., Ali M., Iqbal J., Ali W., Alarifi S., Shinwari Z.K. (2020). Green-Synthesized Silver Nanoparticles Induced Apoptotic Cell Death in MCF-7 Breast Cancer Cells by Generating Reactive Oxygen Species and Activating Caspase 3 and 9 Enzyme Activities. Oxid. Med. Cell. Longev..

[170] Manful C.F., Fordjour E., Subramaniam D., Sey A.A., Abbey L., Thomas R. (2025). Antioxidants and Reactive Oxygen Species: Shaping Human Health and Disease Outcomes. Int. J. Mol. Sci..

[171] Dayem A.A., Hossain M.K., Lee S.B., Kim K., Saha S.K., Yang G.M., Choi H.Y., Cho S.G. (2017). The Role of Reactive Oxygen Species (ROS) in the Biological Activities of Metallic Nanoparticles. Int. J. Mol. Sci..

[172] Cameron S.J., Sheng J., Hosseinian F., Willmore W.G. (2022). Nanoparticle Effects on Stress Response Pathways and Nanoparticle–Protein Interactions. Int. J. Mol. Sci..

[173] Vuković B., Cvetić Ž., Bendelja K., Barbir R., Milić M., Dobrošević B., Šerić V., Vinković Vrček I. (2021). In Vitro Study on the Immunomodulatory Effects of Differently Functionalized Silver Nanoparticles on Human Peripheral Blood Mononuclear Cells. J. Biol. Inorg. Chem..

[174] Liu Y., Hardie J., Zhang X., Rotello V.M. (2017). Effects of Engineered Nanoparticles on the Innate Immune System. Semin. Immunol..

[175] Palacka K., Hermankova B., Cervena T., Rossner P., Zajicova A., Uherkova E., Holan V., Javorkova E. (2025). The Immunomodulatory Effect of Silver Nanoparticles in a Retinal Inflammatory Environment. Inflammation.

[176] Noga M., Milan J., Frydrych A., Jurowski K. (2023). Toxicological Aspects, Safety Assessment, and Green Toxicology of Silver Nanoparticles (AgNPs)—Critical Review: State of the Art. Int. J. Mol. Sci..

[177] Raza K., Kumar P., Kumar N., Malik R. (2017). Pharmacokinetics and Biodistribution of the Nanoparticles. Advances in Nanomedicine for the Delivery of Therapeutic Nucleic Acids.

[178] Salim E.I., Abdel-Halim K.Y., El-Mahalawy M.E., Badr H.A., Ahmed H. (2023). Tissue Distribution, Pharmacokinetics, and Effect of Hematological and Biochemical Parameters of Acute Intravenous Administration of Silver Nanoparticles in Rats. Nanomaterials.

[179] Ferdous Z., Nemmar A. (2020). Health Impact of Silver Nanoparticles: A Review of the Biodistribution and Toxicity Following Various Routes of Exposure. Int. J. Mol. Sci..

[180] Wang K., Wang S., Yin J., Yang Q., Yu Y., Chen L. (2023). Long-Term Application of Silver Nanoparticles in Dental Restoration Materials: Potential Toxic Injury to the CNS. J. Mater. Sci. Mater. Med..

[181] Dąbrowska-Bouta B., Sulkowski G., Strużyński W., Strużyńska L. (2018). Prolonged Exposure to Silver Nanoparticles Results in Oxidative Stress in Cerebral Myelin. Neurotox. Res..

[182] Mishra R.K., Ahmad A., Vyawahare A., Alam P., Khan T.H., Khan R. (2021). Biological effects of formation of protein corona onto nanoparticles. Int. J. Biol. Macromol..

[183] Guo H., Zhang J., Boudreau M., Meng J., Yin J.J., Liu J., Xu H. (2016). Intravenous administration of silver nanoparticles causes organ toxicity through intracellular ROS-related loss of inter-endothelial junction. Part. Fibre Toxicol..

